# Author Correction: Molecular-strain induced phosphinidene reactivity of a phosphanorcaradiene

**DOI:** 10.1038/s41467-024-53231-3

**Published:** 2024-10-11

**Authors:** Yizhen Chen, Peifeng Su, Dongmin Wang, Zhuofeng Ke, Gengwen Tan

**Affiliations:** 1 Key Laboratory of Bioinorganic and Synthetic Chemistry of Ministry of Education, Guangdong Basic Research Center of Excellence for Functional Molecular Engineering, School of Chemistry, IGCME, Sun Yat-sen University, Guangzhou, 510275 China; 2 Innovation Center for Chemical Sciences, Key Laboratory of Organic Synthesis of Jiangsu Province, College of Chemistry, Chemical Engineering and Materials Science, Soochow University, Suzhou, 215123 China; 3 School of Materials Science and Engineering, PCFM Lab, the Key Laboratory of Low-carbon Chemistry & Energy Conservation of Guangdong Province, Sun Yat-sen University, Guangzhou, 510006 China

**Keywords:** Chemical bonding, Ligands, Chemical bonding

Correction to: *Nature Communications* 10.1038/s41467-024-49042-1, published online 29 May 2024

The original version of this Article contained an error in the Results section, which incorrectly read ‘It crystallizes in the monoclinic space group *P*1’. The correct version states ‘It crystallizes in the triclinic space group *P*1’ in place of ‘It crystallizes in the monoclinic space group *P*1’. This has been corrected in both the PDF and HTML versions of the Article.

