# Supplementary material for: Molecular-strain induced phosphinidene reactivity of a phosphanorcaradiene
Source: Nat Commun. 2024 May 29;15:4579. doi: 10.1038/s41467-024-49042-1 (PMC11137065; doi:10.1038/s41467-024-49042-1)
Supplement: Supplementary file 1 — Supplementary Information [file 41467_2024_49042_MOESM1_ESM.pdf]

Supplementary Information

Molecular-strain induced phosphinidene reactivity of a phosphanorcaradiene

Yizhen Chen, Peifeng Su, Dongmin Wang, Zhuofeng Ke,* Gengwen Tan*

Content

Experimental Section....S2

Synthesis of phosphanorcaradiene (**1**)....S3

Synthesis of M^{s}Fluid^{tBu}–PPMe$_3$ (**2**)....S4

Synthesis of M^{s}Fluid^{tBu}-P=C=N-Ar (**3**)....S4

Synthesis of M^{s}Fluid^{tBu}–P=C=N–TMS (**4**)....S5

Synthesis of M^{s}Fluid^{tBu}–P=N–Ar' (**5**)....S6

Synthesis of M^{s}Fluid^{tBu}–P=(N–Ar')$_2$ (**6**)....S6

Synthesis of M^{s}Fluid^{tBu}–P(CH$_2$)CH$_2$ (**7**)....S7

Synthesis of M^{s}Fluid^{tBu}–PCHC–Ar' (**8**)....S8

Synthesis of M^{s}Fluid^{tBu}–PH–SiHEt$_2$ (**9**)....S9

Synthesis of M^{s}Fluid^{tBu}–PH–NHPh (**10**)....S10

Crystal data and refinement of **1-10**....S11

Theoretical Calculations....S14

Selected NMR spectra of **1-10**....S18

References....S33

Experimental Section

General considerations: All experiments were carried out under a dry oxygen-free nitrogen atmosphere using standard Schlenk techniques or in a N_2 filled-glove box. Solvents were dried by standard methods and stored in activated 4 Å molecule sieve in the glovebox. All reagents were purchased from commercial sources (Energy Chemical and TCI) and used without further purification unless otherwise noted. M^sFluind^{tBu}-PCl_2[S1], and KC_8[S2] were synthesized according to reported procedures.

NMR measurements

The ^1H, $^{13}C\{^1H\}$ and $^{31}P\{^1H\}$ NMR spectra were recorded on Bruker spectrometers (AV400 and AV600). Chemical shift values for protons are referenced to the residual proton resonance of $CDCl_3$ (δ: 7.26), C_6D_6 (δ: 7.16), THF-d_8 (δ: 3.62); chemical shift values for carbons are referenced to the carbon resonance of $CDCl_3$ (δ: 77.16), C_6D_6 (δ: 128.06), THF-d_8 (δ: 67.21); chemical shift values for phosphorus are relative to 85% H_3PO_4. NMR multiplicities are abbreviated as follows: s = singlet, d = doublet, t = triplet, q = quartet, sept = septet, m = multiplet, br = broad signal. Chemical shifts are quoted in δ (ppm) and coupling constants in Hz. The samples were dissolved in deuterated solvents, and were sealed off in J-Young NMR tubes for measurements.

Single-crystal X-ray diffraction analyses

For the single crystal X-ray structure analyses the crystals were each mounted on a glass capillary in perfluorinated oil and measured in a cold N_2 flow. The data for all compounds were collected on a Bruker D8 Venture or XtaLAB Synergy R, DW system, HyPix diffractometer at low temperatures. Using Olex2,[S3] the structure was solved with the olex2.solve[S4] structure solution program using Charge Flipping and refined with the SHELXL[S5] refinement package using Least Squares minimisation. The positions of the H atoms were calculated and considered isotropically according to a riding model. Platon SQUEEZE was used to remove the highly disordered solvent molecules in the crystal lattices.[S6]

Synthesis of phosphanorcaradiene (1)

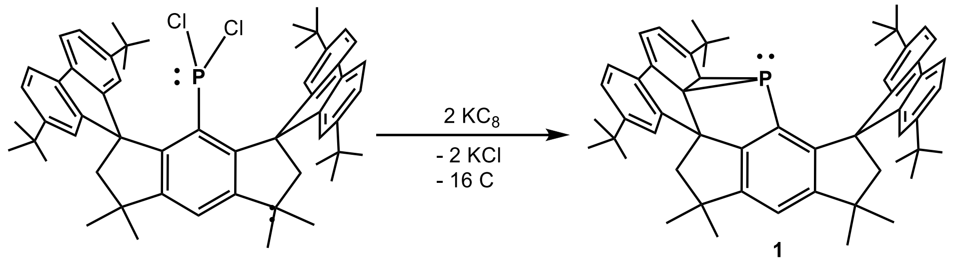

Dry THF (15 ml) was added to a Schlenk flask containing M^{s}Fluid^{tBu}–PCl_2 (0.840 g, 1.0 mmol) and KC_8 (0.284 g, 2.1 mmol) cooled to –78 °C under N_2 atmosphere. The mixture was stirred at –78 °C for 2 h. Subsequently, the mixture was warmed up to –5 °C and stirred at this temperature for 24 h. After filtration, all the dark green suspension was evaporated under vacuum and the residue was extracted with 10 ml of toluene and filtered. The filtrate was concentrated to ca. 5 ml and kept at –30 °C for 12 h to give a pale yellow solid of product **1**. Yield: 0.58 g, 0.75 mmol, 75%. Yellow crystals suitable for X-ray diffraction analysis were obtained from toluene/*n*-hexane solution at –4 °C. ^{1}H NMR (400 MHz, THF-d_8, 298 K, ppm): δ 0.79 (s, 9H, C(C*H*$_3$)$_3$), 1.19 (s, 9H, C(C*H*$_3$)$_3$), 1.33 (s, 9H, C(C*H*$_3$)$_3$), 1.34 (s, 3H, C(C*H*$_3$)$_2$), 1.36 (s, 9H, C(C*H*$_3$)$_3$), 1.56 (s, 3H, C(C*H*$_3$)$_2$), 1.72 (s, 3H, C(C*H*$_3$)$_2$), 1.74 (s, 3H, C(C*H*$_3$)$_2$), 1.89 (d, $^2J_{H,H}$ = 12.8 Hz, 1H, C*H*$_2$), 2.03 (d, $^2J_{H,H}$ = 12.8 Hz, 1H, C*H*$_2$), 2.43 (d, $^2J_{H,H}$ =13.9 Hz, 1H, C*H*$_2$), 2.54 (s, 1H, PC–*H*), 2.64 (d, $^2J_{H,H}$ = 13.9 Hz, 1H, C*H*$_2$), 5.72 (d, $^3J_{H,H}$= 6.4 Hz, 1H, Ar-*H*), 5.99 (d, $^3J_{H,H}$ = 6.4 Hz, 1H, Ar-*H*), 7.10 (s, 1H, Ar-*H*), 7.25 (s, 2H, Ar-*H*), 7.28 (dd, $^3J_{H,H}$ = 7.8 Hz, $^5J_{H,H}$ = 1.7 Hz, 2H, Ar-*H*), 7.33 – 7.37 (m, 2H, Ar-*H*), 7.48 (d, $^4J_{H,H}$ = 2.0 Hz, 1H, Ar-*H*), 7.55 (d, $^3J_{H,H}$ = 7.8 Hz, 1H, Ar-*H*), 7.59 (d, $^3J_{H,H}$ = 8.0 Hz, 1H, Ar-*H*). ^{13}C{^{1}H} NMR (101 MHz, THF-d_8, 298 K, ppm): δ 28.8 (C(*C*H$_3$)$_3$), 29.4 (C(*C*H$_3$)$_2$), 31.6 (C(*C*H$_3$)$_3$), 31.9 (C(*C*H$_3$)$_3$), 33.8 (C(*C*H$_3$)$_2$), 34.4 (C(*C*H$_3$)$_2$), 35.3 (*C*(CH$_3$)$_3$), 35.4 (*C*(CH$_3$)$_3$), 35.5 (*C*(CH$_3$)$_3$), 36.3 (*C*(CH$_3$)$_3$), 44.2 (*C*(CH$_3$)$_2$), 46.2 (P–*C*–H), 50.2 (*C*(CH$_3$)$_2$), 54.7, 56.8 (*C*H$_2$C(CH$_3$)$_2$), 63.4, 63.9 (P–*C*–C–H), 67.8 (*C*CH$_2$C(CH$_3$)$_2$), 111.0, 116.6, 118.9, 119.4, 119.9, 121.2, 121.6, 121.8, 124.2, 124.5, 124.9, 136.7, 138.7, 138.9, 141.4, 144.6, 149.7, 150.3, 151.1 (PC–*C*), 154.6, 155.5 (PCC–*C*), 155.7, 156.3. ^{31}P{^{1}H} NMR (162 MHz, THF-d_8, 298 K, ppm): δ -155.1. Elemental analysis for $C_{56}H_{65}P$ (%): Cacld: C 87.45, H 8.52; Found: C 87.12, H 8.23.

Synthesis of M^{s}Fluid^{tBu}–PPMe$_3$ (2)

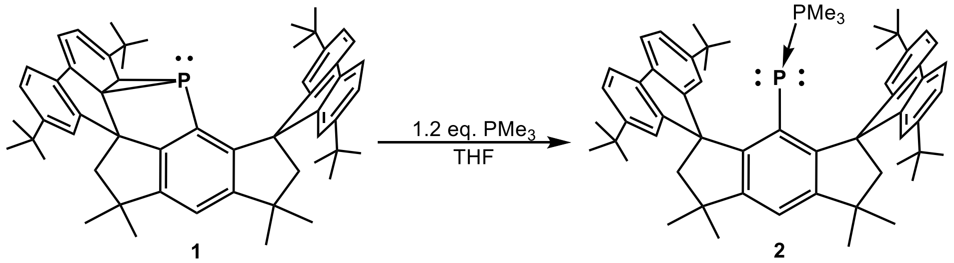

1 **2**

To a solution of **1** (0.385 g, 0.5 mmol) in THF (15 mL), PMe$_3$ (0.6 mL, 0.6 mmol, 1.0 M in THF) was added dropwise and the mixture was stirred at room temperature. After stirring for 6 h, all volatiles were removed under vacuum and the residue was washed with *n*-hexane (5 ml) to yield a yellow solid of **2**. Yield: 0.37 g, 0.44 mmol, 87%. Yellow crystals suitable for X-ray diffraction analysis were obtained from THF/*n*-hexane solution at room temperature. ^{1}H NMR (400 MHz, C_6D_6, 298 K, ppm): δ 0.05 (dd, $^{2}J_{P, H}$ = 12.1 Hz, $^{3}J_{P, H}$ = 2.0 Hz, 9H, P(C$\underline{H}_3$)$_3$), 1.31 (s, 36H, C(C$\underline{H}_3$)$_3$), 1.58 (s, 12H, C(C$\underline{H}_3$)$_2$), 2.46 (s, 4H, C$\underline{H}_2$), 7.30 (d, $^{3}J_{H, H}$ = 8.4 Hz, 4H, Ar-$\underline{H}$), 7.37 (d, $^{4}J_{H, H}$ = 2.8 Hz, 1H, Ar-$\underline{H}$), 7.43 (d, $^{4}J_{H, H}$ = 1.6 Hz, 4H, Ar-$\underline{H}$), 7.61 (d, $^{3}J_{H, H}$ = 7.6 Hz, 4H, Ar-$\underline{H}$). ^{13}C{^{1}H} NMR (101 MHz, C_6D_6, 298 K, ppm): δ 17.8 (P($\underline{C}$H$_3$)$_3$), 32.0 (C($\underline{C}$H$_3$)$_3$), 33.0 (C($\underline{C}$H$_3$)$_2$), 35.0 ($\underline{C}$(CH$_3$)$_3$), 42.4 ($\underline{C}$(CH$_3$)$_2$), 59.7 ($\underline{C}$H$_2$C(CH$_3$)$_2$), 66.6 ($\underline{C}$CH$_2$C(CH$_3$)$_2$), 116.8 (P-Ar-$\underline{C}$H), 119.3, 121.8, 123.4, 127.9, 128.2, 136.5 (P–$\underline{C}$–C), 138.4, 149.6, 153.5 (PC–$\underline{C}$–C), 155.4, 155.5, 156.6. ^{31}P{^{1}H} NMR (162 MHz, C_6D_6, 298 K, ppm): δ -0.9 (d, $^{1}J_{P, P}$ = 603 Hz, P–$\underline{P}$(CH$_3$)$_3$), -157.8 (d, $^{1}J_{P, P}$ = 603 Hz, $\underline{P}$–P(CH$_3$)$_3$). Elemental analysis for $C_{59}H_{74}P_2$ (%): Cacld: C 83.85, H 8.83; Found: C 83.23, H 8.51.

Synthesis of M^{s}Fluid^{tBu}-P=C=N-Ar (3)

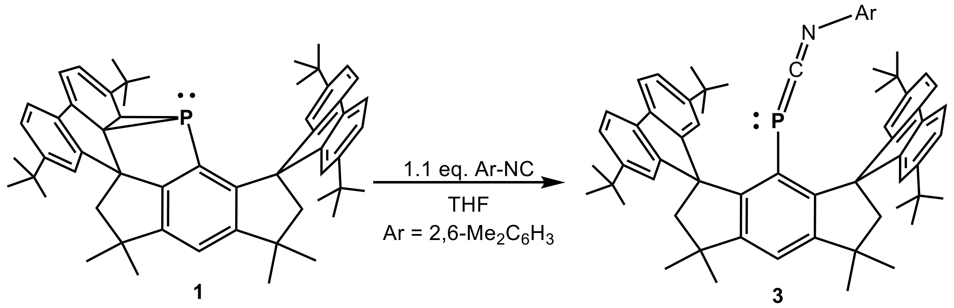

1 **3**

2,6-dimethylphenyl isocyanide (0.072 g, 0.55 mmol) was added to a solution of **1** (0.385 g, 0.5 mmol) in THF (15 ml) at room temperature. The mixture was stirred for further 10 h. After removal of all volatiles

under vacuum, the residue was extracted with 15 ml of *n*-hexane, and filtered over Celite. The filtrate was concentrated to ca. 8 ml and left at –30 °C overnight to give an orange solid of **3**. Yield: 0.34 g, 0.38 mmol, 75%. Yellow crystals suitable for X-ray diffraction analysis were obtained from toluene/*n*-hexane solution at –20 °C. ^{1}H NMR (400 MHz, C_6D_6, 298 K, ppm): δ 1.26 (s, 36H, C(C$\underline{H}_3$)$_3$), 1.53 (s, 6H, Ar–C$\underline{H}_3$), 1.61 (s, 12H, C(C$\underline{H}_3$)$_2$), 2.52 (s, 4H, C$\underline{H}_2$), 6.58 (d, $^{3}J_{H,H}$ = 7.5 Hz, 2H, Ar-$\underline{H}$), 6.66 (t, $^{3}J_{H,H}$ = 7.5 Hz, 1H, Ar-$\underline{H}$), 7.14 (d, $^{3}J_{H,H}$ = 9.6 Hz, 4H, Ar-$\underline{H}$), 7.37 (s, 4H, Ar-$\underline{H}$), 7.38 (s, 1H, Ar-$\underline{H}$), 7.47 (d, $^{3}J_{H,H}$ = 8.0 Hz, 4H, Ar-$\underline{H}$). ^{13}C{^{1}H} NMR (101 MHz, C_6D_6, 298 K, ppm): δ 19.4 (Ar–$\underline{C}$H$_3$), 31.9 (C($\underline{C}$H$_3$)$_3$), 33.1 (C($\underline{C}$H$_3$)$_2$), 35.0 ($\underline{C}$(CH$_3$)$_3$), 42.8 ($\underline{C}$(CH$_3$)$_2$), 58.7 ($\underline{C}$H$_2$C(CH$_3$)$_2$), 65.4 ($\underline{C}$CH$_2$C(CH$_3$)$_2$), 117.7, 119.6, 121.1, 124.0, 125.5, 127.9, 131.9, 138.7, 150.0, 150.4, 154.5, 155.7, 184.8 (P=$\underline{C}$=N). ^{31}P{^{1}H} NMR (162 MHz, C_6D_6, 298 K, ppm): δ -152.3. Elemental analysis for $C_{65}H_{74}NP$ (%): Cacld: C 86.72, N 1.56, H 8.29; Found: C 86.15, N 1.43, H 8.13.

Synthesis of M^{s}Fluid^{tBu}–P=C=N–TMS (4)

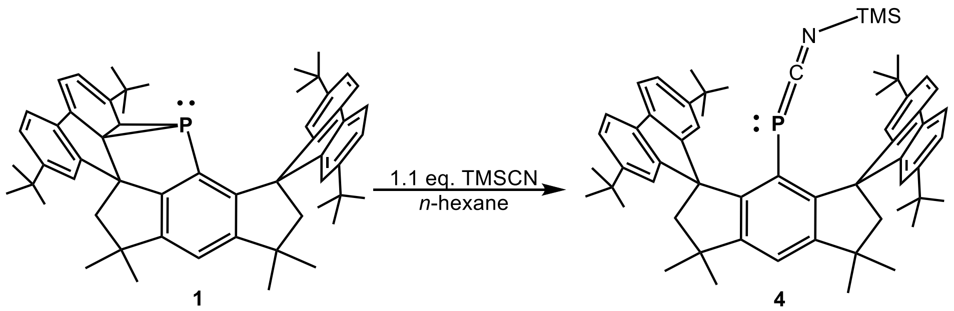

1 4

Compound **1** (0.385 g, 0.5 mmol) and trimethylsilanecarbonitrile (0.054 g, 0.55 mmol) were placed in a Schlenk flask (50 ml), and *n*-hexane (15 ml) was added in a nitrogen-filled glovebox at room temperature. The mixture was allowed to stir for 48 h, and a red solution was formed. The solution was concentrated to ca. 5 ml and left at -30 °C for 12 h affording deep red crystals of **4**. Yield: 0.31 g, 0.36 mmol, 71%. ^{1}H NMR (400 MHz, C_6D_6, 298 K, ppm): δ -0.41 (s, 9H, Si(C$\underline{H}_3$)$_3$), 1.30 (s, 36H, C(C$\underline{H}_3$)$_3$), 1.60 (s, 12H, C(C$\underline{H}_3$)$_2$), 2.54 (s, 4H, C$\underline{H}_2$), 7.25 (dd, $^{3}J_{H,H}$ = 7.9 Hz, $^{4}J_{H,H}$ = 1.8 Hz, 4H, Ar-$\underline{H}$), 7.35 (s, 1H, Ar-$\underline{H}$), 7.35 (s, 4H, Ar-$\underline{H}$), 7.58 (d, $^{3}J_{H,H}$ = 7.9 Hz, 4H, Ar-$\underline{H}$). ^{13}C{^{1}H} NMR (101 MHz, C_6D_6, 298 K, ppm): δ -0.5 (Si($\underline{C}$H$_3$)$_3$), 32.0 (C($\underline{C}$H$_3$)$_3$), 33.0 (C($\underline{C}$H$_3$)$_2$), 35.0 ($\underline{C}$(CH$_3$)$_3$), 42.8 ($\underline{C}$(CH$_3$)$_2$), 58.3 ($\underline{C}$H$_2$C(CH$_3$)$_2$), 65.4 ($\underline{C}$CH$_2$C(CH$_3$)$_2$), 117.1, 119.2, 121.3, 123.7, 139.0, 149.9, 155.2, 179.5 (P=$\underline{C}$=N). ^{31}P{^{1}H} NMR (162 MHz, C_6D_6, 298 K, ppm): δ -190.5. Elemental analysis for $C_{60}H_{74}NPSi$ (%): Cacld: C 82.99, N 1.61, H

8.59; Found: C 82.45, N 1.36, H 8.21.

Synthesis of M^{s}Fluid^{tBu}–P=N–Ar' (5)

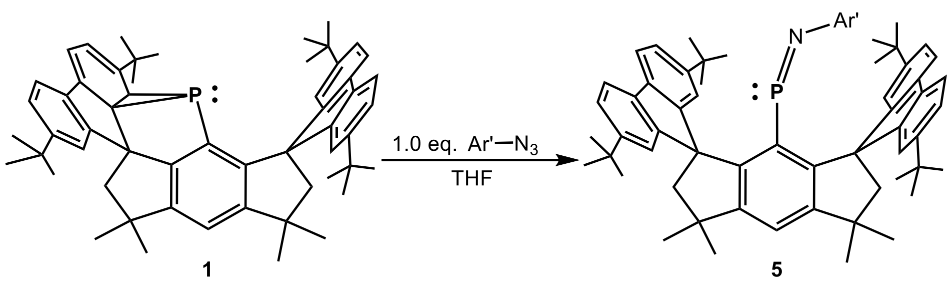

1 **5**

To a solution of **1** (0.385 g, 0.5 mmol) in dry THF (20 ml) was added to 1-azido-4-tert-butylbenzene solution (0.1M in THF, 5 ml, 0.5 mmol) at room temperature. After stirring for 2 h, all the volatiles were removed under vacuum. The residue was extracted with diethyl ether (10 × 2 ml) and filtered. The filtrate was concentrated to ca. 5 ml and kept at –40 °C for 24 h to give orange solid. The crude product was washed with *n*-hexane (5 ml) to obtain **5** as red solid. Yield: 0.15 g, 0.16 mmol, 33%. ^{1}H NMR (400 MHz, C_6D_6, 298 K, ppm): δ 1.15 (s, 9H, Ar'–C(C*H*$_3$)$_3$), 1.24 (s, 36H, C(C*H*$_3$)$_3$), 1.61 (s, 12H, C(C*H*$_3$)$_2$), 2.51 (s, 4H, C*H*$_2$), 5.97 (d, $^3J_{H,H}$ = 8.3 Hz, 2H, Ar'-*H*), 7.01 (d, $^3J_{H,H}$ = 8.2 Hz, 2H, Ar'-*H*), 7.15 (d, $^3J_{H,H}$ = 7.8 Hz, 4H, Ar-*H*), 7.34 (d, $^3J_{H,H}$ = 7.9 Hz, 4H, Ar-*H*), 7.41 (s, 1H, Ar-*H*), 7.44 (s, 4H, Ar-*H*). ^{13}C{^{1}H} NMR (101 MHz, C_6D_6, 298 K, ppm): δ 31.6 (Ar'-C(*C*H$_3$)$_3$), 31.8 (C(*C*H$_3$)$_3$), 32.9 (C(*C*H$_3$)$_2$), 33.0 (Ar'-*C*(CH$_3$)$_3$), 35.0 (*C*(CH$_3$)$_3$), 43.7 (*C*(CH$_3$)$_2$), 57.8 (*C*H$_2$C(CH$_3$)$_2$), 63.6 (*C*CH$_2$C(CH$_3$)$_2$), 119.7, 121.9, 124.4, 124.6, 137.9, 147.6, 147.7, 150.5, 154.3, 155.0, 155.3, 155.6. ^{31}P{^{1}H} NMR (162 MHz, C_6D_6, 298 K, ppm): δ 424.2. Elemental analysis for $C_{66}H_{78}NP$ (%): Cacld: C 86.51, H 8.58, N 1.53; Found: C 86.82, H 8.23, N 1.67.

Synthesis of M^{s}Fluid^{tBu}–P=(N–Ar')$_2$ (6)

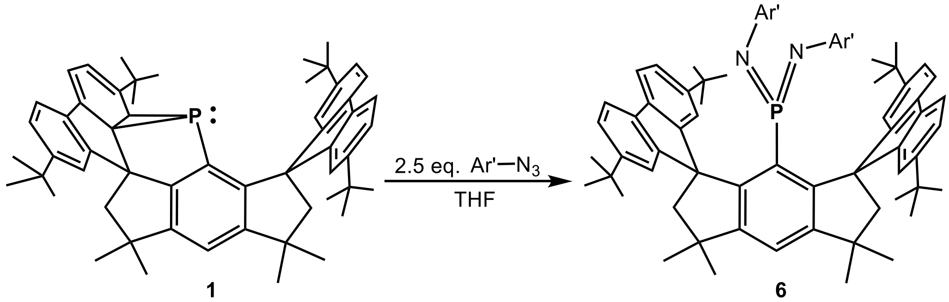

1 **6**

In a N_2-filled glovebox, 1-azido-4-tert-butylbenzene (0.22 g, 1.25 mmol) was added to a solution of **1** (0.385 g, 0.5 mmol) in THF (20 ml) at room temperature. The mixture solution was stirred for 24 h and the volatiles were removed under vacuum to give yellow residue, which was ubsequently washed with small portions of *n*-hexane (total volume of 5 ml) to **6** as a yellow solid. Yield: 0.36 g, 0.34 mmol, 68%. Yellow crystals suitable for X-ray diffraction analysis were obtained from toluene solution at –30 °C. ^{1}H NMR (600 MHz, C_6D_6, 298 K, ppm): δ 1.16 (s, 36H, C(C$\underline{H}_3$)$_3$), 1.16 (s, 18H, Ar'–C(C$\underline{H}_3$)$_3$), 1.54 (s, 12H, C(C$\underline{H}_3$)$_2$), 2.50 (s, 4H, C$\underline{H}_2$), 5.51 (d, $^3J_{H,H}$ = 8.5 Hz, 4H, Ar'-$\underline{H}$), 6.83 (d, $^3J_{H,H}$ = 8.0 Hz, 4H, Ar'-$\underline{H}$), 7.23 (d, $^3J_{H,H}$ = 7.9 Hz, 4H, Ar-$\underline{H}$), 7.44 (s, 4H, Ar-$\underline{H}$), 7.52 (d, $^3J_{H,H}$ = 7.9 Hz, 4H, Ar-$\underline{H}$), 7.56 (s, 1H, Ar-$\underline{H}$). ^{13}C{^{1}H} NMR (151 MHz, C_6D_6, 298 K, ppm): δ 31.6 (C($\underline{C}$H$_3$)$_3$), 32.5 (Ar'–C($\underline{C}$H$_3$)$_3$), 34.1 (Ar'–$\underline{C}$(CH$_3$)$_3$), 34.9 ($\underline{C}$(CH$_3$)$_3$), 42.7 ($\underline{C}$(CH$_3$)$_2$), 59.7 ($\underline{C}$H$_2$C(CH$_3$)$_2$), 64.8 ($\underline{C}$CH$_2$C(CH$_3$)$_2$), 119.7 122.0, 123.8, 124.9, 126.0, 138.9, 141.6, 144.1, 144.7, 150.4, 153.4, 155.6, 156.4 (PN–$\underline{C}$(^{t}BuPh)). ^{31}P{^{1}H} NMR (243 MHz, C_6D_6, 298 K, ppm): δ 45.1. Elemental analysis for $C_{76}H_{91}N_2P$ (%): Cacld: C 85.83, H 8.62, N 2.63; Found: C 85.25, H 8.97, N 2.79.

Synthesis of M^{s}Fluid^{tBu}–P(CH$_2$)CH$_2$ (7)

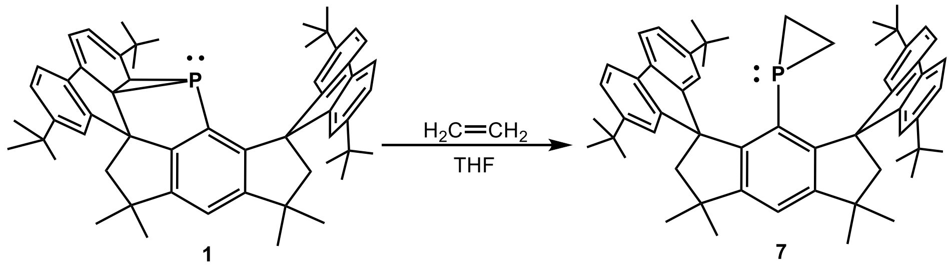

A solution of **1** (0.385 g, 0.5 mmol) in THF (20 ml) was frozen in the liquid nitrogen bath and degassed. Ethylene (1 atm.) was introduced, and the mixture was stirred for 24 h at room temperature. All the volatiles were removed under vacuum, and the residue was washed with THF (5 ml) to give a white solid of **7**. Yield: 0.26 g, 0.33 mmol, 65%. Colorless crystals of suitable for SC-XRD analysis were formed from a THF/hexane solution at room temperature. ^{1}H NMR (400 MHz, $CDCl_3$, 298 K, ppm): δ -0.95 – -0.82 (m, 4H, P(C$\underline{H}_2$)$_2$), 1.23 (s, 36H, C(C$\underline{H}_3$)$_3$), 1.56 (s, 12H, C(C$\underline{H}_3$)$_2$), 2.40 (s, 4H, C$\underline{H}_2$), 7.12 (d, $^4J_{H,H}$ = 2.0 Hz, 4H, Ar-$\underline{H}$), 7.22 (dd, $^3J_{H,H}$ = 7.9 Hz, $^4J_{H,H}$ = 1.9 Hz, 4H, Ar-$\underline{H}$), 7.28 (s, 1H, Ar-$\underline{H}$), 7.47 (d, $^3J_{H,H}$ = 8.0 Hz, 4H, Ar-$\underline{H}$). ^{13}C{^{1}H} NMR (101 MHz, $CDCl_3$, 298 K, ppm): δ 7.2 (P–$\underline{C}$H$_2$), 31.9 (C($\underline{C}$H$_3$)$_3$), 32.9 (C($\underline{C}$H$_3$)$_2$), 35.0 ($\underline{C}$(CH$_3$)$_3$), 42.9 ($\underline{C}$(CH$_3$)$_2$), 58.5 ($\underline{C}$H$_2$C(CH$_3$)$_2$), 64.7 ($\underline{C}$CH$_2$C(CH$_3$)$_2$), 68.2

(*C*CH$_2$C(CH$_3$)$_2$), 117.9, 118.9, 121.3, 123.7, 138.2, 150.2, 154.6, 155.4. ^{31}P{^{1}H} NMR (162 MHz, CDCl$_3$, 298 K, ppm): δ -229.2. Elemental analysis for $C_{58}H_{69}P$ (%): Cacld: C 87.39, H 8.72; Found: C 86.96, H 8.42.

Synthesis of M^{s}Fluid^{tBu}–PCHC–Ar' (8)

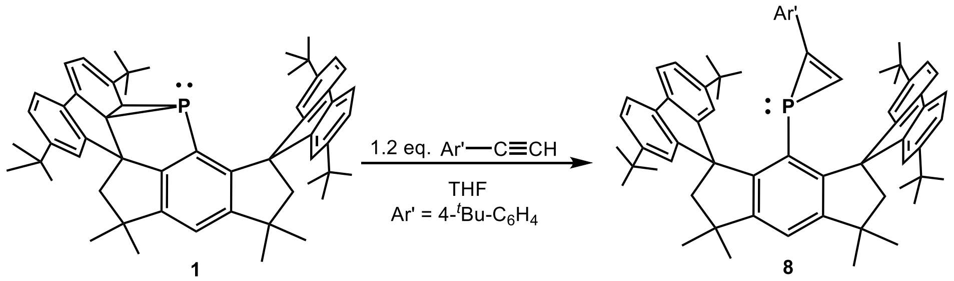

1 **8**

The solution of **1** (0.385 g, 0.5 mmol) and 1-(*tert*-butyl)-4-ethynylbenzene (0.095 g, 0.6 mmol) in anhydrous THF was stirred overnight and all the volatiles were removed under vacuum. The residue was extracted with toluene (15 ml) and filtered. The filtrate was concentrated to ca. 5 ml and kept at –40 °C for 24 h to afford **8** as colorless crystals. Yield: 0.30 g. 0.32 mmol, 65%. Colorless crystals of suitable for SC-XRD analysis were formed from a toluene solution at –30 °C. ^{1}H NMR (400 MHz, C_6D_6, 298 K, ppm): δ 1.24 (s, 9H, Ar-C(C*H*$_3$)$_3$), 1.25 (s, 18H, C(C*H*$_3$)$_3$), 1.35 (s, 18H, C(C*H*$_3$)$_3$), 1.51 (s, 6H, C(C*H*$_3$)$_2$), 1.54 (s, 6H, C(C*H*$_3$)$_2$), 2.48 (s, 4H, C*H*$_2$), 5.44 (d, $^2J_{P,H}$ = 22.6 Hz, 1H, P–C–*H*), 6.13 (d, $^3J_{H,H}$ = 8.2 Hz, 2H, Ar'-*H*), 6.97 (d, $^3J_{H,H}$ = 8.2 Hz, 2H, Ar'-*H*), 7.22 – 7.27 (m, 4H, Ar-*H*), 7.37 (s, 1H, Ar-*H*), 7.42 (s, 2H, Ar-*H*), 7.45 (d, $^3J_{H,H}$ = 8.0 Hz, 2H, Ar-*H*) 7.47 (s, 2H, Ar-*H*), 7.55 (d, $^3J_{H,H}$ = 7.9 Hz, 2H, Ar-*H*). ^{13}C{^{1}H} NMR (101 MHz, C_6D_6, 298 K, ppm): δ 31.1 (Ar'–C(*C*H$_3$)$_3$), 31.6 (C(*C*H$_3$)$_3$), 32.2 (C(*C*H$_3$)$_2$), 34.2 (Ar'–*C*(CH$_3$)$_3$), 34.6 (*C*(CH$_3$)$_3$), 42.2 (*C*(CH$_3$)$_2$), 60.3 (*C*H$_2$C(CH$_3$)$_2$), 64.7 (*C*CH$_2$C(CH$_3$)$_2$), 117.0 (P–*C*–H), 117.3, 119.5 (C(CH$_3$)$_3$–*C*H–CH), 120.9, 121.0, 123.3, 123.6, 124.3 (Ar'-*C*H), 128.1 (Ar'-*C*H), 134.2 (P–C–*C*(Ar')), 137.4, 137.5, 140.4, 141.4, 147.0, 149.7, 150.2, 156.2, 156.33. ^{31}P{^{1}H} NMR (162 MHz, C_6D_6, 298 K, ppm): δ -171.3. Elemental analysis for $C_{68}H_{79}P$ (%): Cacld: C 88.07, H 8.59; Found: C 87.87, H 8.82.

Synthesis of M^sFluid^{tBu}–PH–SiHEt₂ (9)

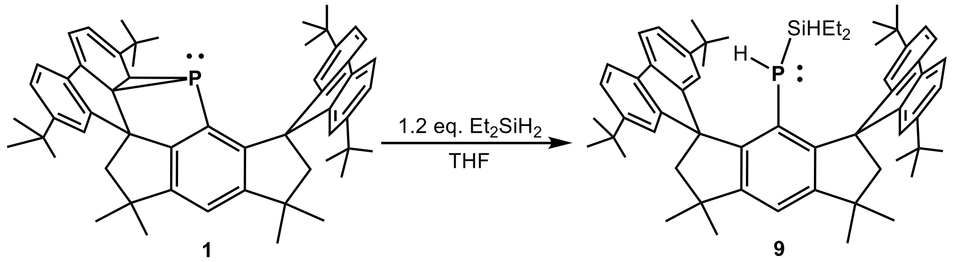

1 **9**

Diethylsilane (0.053 g, 0.6 mmol) was added to a solution of **1** (0.384 g, 0.5 mmol) in THF (20 ml) at room temperature and stirred for 12 h. After removal of all volatiles under vacuum, the residue was extracted with toluene (10 ml × 2) and filtered. The filtrate was concentrated to ca. 8 ml and kept at –30 °C for 24 h to give **9** as colorless crystals. Yield: 0.30 g. 0.35 mmol, 70%. Colorless crystals of suitable for SC-XRD analysis were formed from a C_6D_6/*n*-hexane solution at room temperature. ^{1}H NMR (400 MHz, C_6D_6, 298 K, ppm): δ -0.86 – -0.75 (m, 1H, SiH–C$\underline{H}_2$), -0.56 – -0.47 (m, 2H, SiH–C$\underline{H}_2$), -0.36 – -0.24 (m, 1H, SiH–C$\underline{H}_2$), 0.39 (td, $^3J_{H,H}$ = 7.8 Hz, $^4J_{H,H}$ = 2.1 Hz, 6H, SiH–CH$_2$–C$\underline{H}_3$), 1.30 (s, 18H, C(C$\underline{H}_3$)$_3$), 1.32 (s, 18H, C(C$\underline{H}_3$)$_3$), 1.53 (s, 6H, C(C$\underline{H}_3$)$_2$), 1.57 (s, 6H, C(C$\underline{H}_3$)$_2$), 2.26 (dd, $^1J_{P,H}$ = 219.2 Hz, $^3J_{H,H}$ = 3.2 Hz, 1H, SiH–P$\underline{H}$), 2.43 (d, $^2J_{H,H}$ = 13.7 Hz, C$\underline{H}_2$), 2.50 (d, $^2J_{H,H}$ = 13.7 Hz, C$\underline{H}_2$), 2.72 (d, $^2J_{P,H}$ = 18.6 Hz, $^3J_{H,H}$ = 2.9 Hz, P–Si–$\underline{H}$), 7.30 – 7.32 (m, 3H, Ar-$\underline{H}$), 7.33 (s, 2H, Ar-$\underline{H}$), 7.40 (s, 1H, Ar-$\underline{H}$), 7.46 (s, 1H, Ar-$\underline{H}$), 7.62 (dd, $^3J_{H,H}$ = 8.0 Hz, $^4J_{H,H}$ = 3.2 Hz, 4H, Ar-$\underline{H}$). ^{13}C{^{1}H} NMR (101 MHz, C_6D_6, 298 K, ppm): δ 3.2 (Si–$\underline{C}$H$_2$), 4.0 (Si–$\underline{C}$H$_2$), 8.0 (Si–$\underline{C}$H$_2$), 8.5 (SiCH$_2$–$\underline{C}$H$_3$), 31.5 (C($\underline{C}$H$_3$)$_3$), 32.4 (C($\underline{C}$H$_3$)$_2$), 34.7 ($\underline{C}$(CH$_3$)$_3$), 42.0 ($\underline{C}$(CH$_3$)$_2$), 59.9 ($\underline{C}$H$_2$C(CH$_3$)$_2$), 65.2 ($\underline{C}$CH$_2$C(CH$_3$)$_2$), 116.0, 119.5, 121.1, 121.7, 124.1, 130.8, 131.2, 137.6, 146.3, 149.9, 150.0, 153.6, 154.7, 156.0. ^{31}P{^{1}H} NMR (162 MHz, C_6D_6, 298 K, ppm): δ -164.7. Elemental analysis for $C_{60}H_{77}PSi$ (%): Cacld: C 84.06, H 9.05; Found: C 83.69, H 8.76.

Synthesis of $M^{s}Fluid^{tBu}$–PH–NHPh (10)

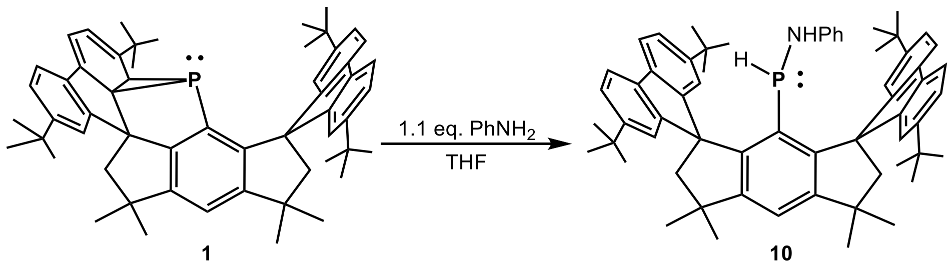

1 **10**

Aniline (0.051 g, 0.55 mmol) was added to a solution of **1** (0.384 g, 0.5 mmol) in THF (20 ml) at room temperature and stirred for 12 h. After removal of all volatiles under vacuum, the residue was extracted with toluene (10 ml × 2) and filtered. The filtrate was concentrated to ca. 5 ml and kept at –40 °C for 24 h to give **10** as a white solid. Yield: 0.32 g. 0.37 mmol, 74%. Colorless crystals of suitable for SC-XRD analysis were formed from a toluene/*n*-hexane solution at room temperature. ^{1}H NMR (600 MHz, $CDCl_3$, 298 K, ppm): δ 1.03 (s, 18H, $C(C\underline{H}_3)_3$), 1.27 (s, 18H, $C(C\underline{H}_3)_3$), 1.59 (s, 6H, $C(C\underline{H}_3)_2$), 1.61 (s, 6H, $C(C\underline{H}_3)_2$), 2.39 (d, $^2J_{H,\,H}$ = 13.9 Hz, 2H, $C\underline{H}_2$), 2.40 (d, $^2J_{P,\,H}$ = 59.7 Hz, 1H, N$\underline{H}$), 2.44 (d, $^2J_{H,\,H}$ = 13.9 Hz, 2H, $C\underline{H}_2$), 4.17 (dd, $^1J_{P,\,H}$ = 229.7 Hz, $^3J_{H,\,H}$ = 3.7 Hz, 1H, NH–P$\underline{H}$), 5.24 (d, $^3J_{H,\,H}$ = 7.8 Hz, 2H, Ph-$\underline{H}$), 6.30 (t, $^3J_{H,\,H}$ = 7.2 Hz, 1H, Ph-$\underline{H}$), 6.55 (t, $^3J_{H,\,H}$ = 7.6 Hz, 2H, Ph-$\underline{H}$), 7.02 (s, 2H, Ar-$\underline{H}$), 7.06 (d, $^3J_{H,\,H}$ = 7.8 Hz, 2H, Ar-$\underline{H}$), 7.17 (s, 2H, Ar-$\underline{H}$), 7.33 (d, $^3J_{H,\,H}$ = 7.9 Hz, 2H, Ar-$\underline{H}$), 7.40 (s, 1H, Ar-$\underline{H}$), 7.42 (d, $^3J_{H,\,H}$ = 7.8 Hz, 2H, Ar-$\underline{H}$), 7.55 (d, $^3J_{H,\,H}$ = 7.9 Hz, 2H, Ar-$\underline{H}$),. ^{13}C{^{1}H} NMR (151 MHz, $CDCl_3$, 298 K, ppm): δ 31.7 ($C(\underline{C}H_3)_3$), 32.9 ($C(\underline{C}H_3)_2$), 35.0 ($\underline{C}(CH_3)_3$), 42.8 ($\underline{C}(CH_3)_2$), 59.0 ($\underline{C}H_2C(CH_3)_2$), 64.6 ($\underline{C}CH_2C(CH_3)_2$), 115.3, 116.8, 119.4, 121.0, 124.0, 127.8, 128.9, 131.1, 136.9, 137.5, 146.8, 149.2, 150.6, 155.6, 156.3, 156.5. ^{31}P{^{1}H} NMR (243 MHz, $CDCl_3$, 298 K, ppm): δ -31.1. Elemental analysis for $C_{62}H_{72}NP$ (%): Cacld: C 86.37, N 1.62, H 8.42; Found: C 85.85, N 1.79, H 8.76.

Supplementary Table 1. Crystal data and refinement of 1-10

	**1**	**2**	**3**
formula	$C_{56}H_{65}P$	$C_{59}H_{74}P_2$	$C_{65}H_{74}NP$
formula weight	769.05	845.12	900.22
crystal system	Triclinic	Triclinic	Triclinic
space group	*P*1	*P*-1	*P*-1
a/Å	10.6563(4)	12.5242(13)	11.710(3)
b/Å	14.9602(6)	15.3954(19)	15.493(3)
c/Å	16.9854(7)	16.2667(19)	16.949(3)
α/deg	109.379(2)	63.680(4)	67.011(7)
β/deg	98.437(2)	68.606(4)	70.279(7)
γ/deg	107.495(2)	76.450(4)	77.343(7)
V/Å^3	2343.04(17)	2607.7(5)	2651.2(10)
Z	2	2	2
ρ_{calcd}/g·cm^{-3}	1.090	1.076	1.128
μ/mm^{-1}	0.495	0.118	0.092
F(000)	832.0	916	972
crystal size/mm^3	0.20 × 0.20 × 0.16	0.20 × 0.15 × 0.12	0.20 × 0.15 × 0.15
θ range/deg	4.988–108.09	3.506–54.206	4.46–54.206
index ranges	-12 ≤ h ≤ 12	-15 ≤ h ≤ 16	-15 ≤ h ≤ 15
	-18 ≤ k ≤ 18	-19 ≤ k ≤ 19	-19 ≤ k ≤ 19
	-20 ≤ l ≤ 20	-20 ≤ l ≤ 20	-21 ≤ l ≤ 21
collected data	15676	11408	11664
unique data	13173	7940	8602
	(R_{int} = 0.0499)	(R_{int} = 0.0643)	(R_{int} = 0.0836)
completeness to *θ*	99.5%	99.2%	99.8%
GOF on F^2	1.042	1.048	1.046
final *R* indices	R_1 = 0.0590	R_1 = 0.0959	R_1 = 0.0609
[*I*>2□(*I*)]	wR_2 = 0.1483	wR_2 = 0.2510	wR_2 = 0.1434
R indices (all data)	R_1 = 0.0783	R_1 = 0.1312	R_1 = 0.0858
	wR_2 = 0.1596	wR_2 = 0.2834	wR_2 = 0.1610
Largest diff. peak/hole (e·Å^{-3})	0.96/-0.50	1.96/-0.83	0.53/–0.31

	**4**	**6**	**7**
formula	$C_{60}H_{74}NPSi$	$C_{97}H_{115}N_2P$	$C_{61}H_{76}P$
formula weight	868.26	1339.87	840.18
crystal system	Triclinic	Triclinic	Monoclinic
space group	P-1	P-1	$P2_1$/c
a/Å	12.3061(5)	11.25906(14)	14.8658(2)
b/Å	13.1652(6)	15.31234(17)	16.0329(2)
c/Å	16.8346(7)	25.3457(3)	21.3855(2)
α/deg	87.941(3)	77.1555(9)	90
β/deg	89.107(3)	81.7099(9)	98.6170(10)
γ/deg	81.387(3)	71.0079(11)	90
V/Å^3	2694.8(2)	4016.16(8)	5039.52(10)
Z	2	2	4
ρ_{calcd}/g·cm^{-3}	1.070	1.108	1.107
μ/mm^{-1}	0.925	0.648	0.747
F(000)	940	1452	1828
crystal size/mm^3	0.16 × 0.12 × 0.08	0.25 × 0.20 × 0.10	0.153 × 0.116 × 0.05
θ range/deg	5.254–155.566	6.214–155.628	6.014–134.962
index ranges	-11 ≤ h ≤ 15	-14 ≤ h ≤ 12	-17 ≤ h ≤ 16
	-16 ≤ k ≤ 16	-19 ≤ k ≤ 19	-19 ≤ k ≤ 19
	-19 ≤ l ≤ 21	-31 ≤ l ≤ 31	-24 ≤ l ≤ 25
collected data	10981	16679	9049
unique data	7443	14801	7617
	(R_{int} = 0.0737)	(Rint = 0.0441)	(R_{int} = 0.0424)
completeness to θ	99.4%	100%	99.7%
GOF on F^2	1.050	1.024	1.035
final R indices	R_1 = 0.0688	R_1 = 0.0458	R_1 = 0.0654
[I>2□(I)]	wR_2 = 0. 1814	wR_2 = 0.1186	wR_2 = 0.1699
R indices (all data)	R_1 = 0.1063	R_1 = 0.0514	R_1 = 0.0769
	wR_2 = 0. 2021	wR_2 = 0.1224	wR_2 = 0.1770
Largest diff. peak/hole (e·Å^{-3})	0.71/–0.43	0.64/-0.47	2.80/-0.90

	**8**	**9**	**10**
formula	$C_{68}H_{79}P$	$C_{63}H_{80}PSi$	$C_{62}H_{72}NP$
formula weight	927.28	896.33	862.17
crystal system	Monoclinic	Monoclinic	Monoclinic
space group	$P2_1$/c	$P2_1$/c	$P2_1$/c
a/Å	15.1709(10)	15.1306(3)	15.0645(3)
b/Å	17.3231(11)	18.8158(4)	18.5226(4)
c/Å	21.2349(14)	19.7509(5)	19.6454(4)
α/deg	90	90	90
β/deg	98.791(3)	106.7650(10)	111.690(2)
γ/deg	90	90	90
V/Å^3	5515.1(6)	5384.0(2)	5093.61(19)
Z	4	4	4
ρ_{calcd}/g·cm^{-3}	1.117	1.106	1.124
μ/mm^{-1}	0.471	0.934	0.76
F(000)	2008	1948	1864
crystal size/mm^3	0.10 × 0.08 × 0.06	0.20 × 0.15 × 0.10	0.12 × 0.10 × 0.06
θ range/deg	5.128–118.97	6.1–137.098	6.314–151.582
index ranges	$-19 \le h \le 19$	$-18 \le h \le 17$	$-18 \le h \le 15$
	$-22 \le k \le 22$	$-22 \le k \le 22$	$-22 \le k \le 22$
	$-27 \le l \le 27$	$-23 \le l \le 23$	$-24 \le l \le 24$
collected data	12216	9871	10111
unique data	7673	8610	7575
	(R_{int} = 0.0705)	(R_{int} = 0.0645)	(Rint = 0.0691)
completeness to θ	100%	100%	99.6%
GOF on F^2	1.060	1.044	0.906
final R indices	R_1 = 0.0852	R_1 = 0.0485	R_1 = 0.0724
[I>2□(I)]	wR_2 = 0.2351	wR_2 = 0.1439	wR_2 = 0.1920
R indices (all data)	R_1 = 0.1252	R_1 = 0.0550	R_1 = 0.0975
	wR_2 = 0.2709	wR_2 = 0.1501	wR_2 = 0.2101
Largest diff. peak/hole (e·Å^{-3})	1.37/-0.86	0.37/-0.47	0.38/-0.77

Theoretical Calculations

Optimization of all the structures involved in this study in the gas phase was performed by DFT with the BP86-D3BJ functional using the def2-SVP basis set.[S7-S11] Based on the optimized structures, frequency analysis calculations were carried out to confirm whether the structures were at minima (no imaginary frequency) or transition states (only one imaginary frequency). Intrinsic reaction coordinate (IRC) computations were performed to confirm that related reactants and products were connected by the transition states. Further refinement of the energy results based on the BP86-D3/def2-SVP optimized geometries was conducted by calculating the single-point energy in tetrahydrofuran (ε=7.4) at the BP86-D3/def2-TZVPP/SMD level of theory to consider the solvent effect.[S7-S12] Half-entropy corrections on the energy results were conducted to avoid the overestimation of the entropic contribution due to the effect of solvent. The distortion analyses were performed at the BP86-D3/def2-TZVPP level of theory.[S7-S11] Gaussian 16 software package was employed for all the DFT computations.[S13] The CYLview visualization program was utilized for the 3D depiction of the optimized structure.[S14] The Wiberg bond index, quantum theory of atoms in molecules (QTAIM) analyses[S15] and electron localization function (ELF) analyses[S16] were performed using Multiwfn (Version 3.8).[S17] The wavefunction files for QTAIM were obtained from Gaussian 16 at the same level of theory. Besides, the natural bond orbital (NBO) analysis[S18] was performed at the BP86-D3BJ/Def2SVP level. Intrinsic bond orbital (IBOs)[S19] computations were performed with ORCA program[S20] at the M062X/def2-TZVP level of theory and visualized by IBOview program. Percent buried volume analysis was performed with MORFEUS based on SambVca 2.1 web application.[S21-S22]

a) b)

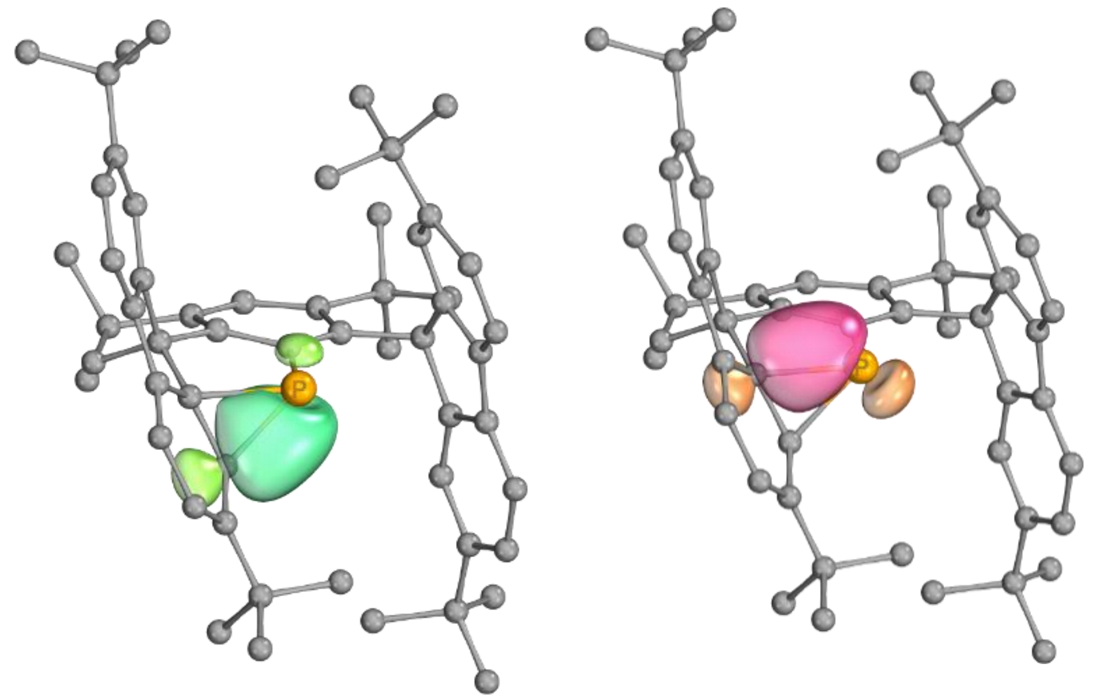

Supplementary Fig. 1 Selected IBOs of **1**. (a) Twisted P1–C27 σ-bonding orbitals. (b) Twisted P1–C28 σ-bonding orbitals.

a) b)

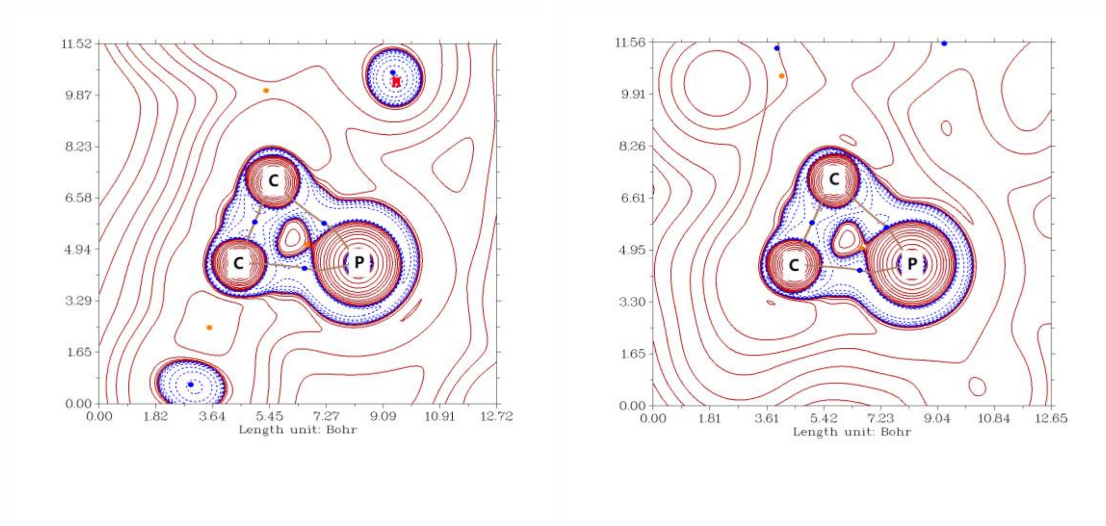

Supplementary Fig. 2 Plot of the Laplacian of the electron density on the C_2P ring, with bond paths (light grey lines) and BCPs (blue dots). (a) the C_2P plane of **1**. (b) the C_2P plane of the phosphanorcaradiene reported by Stephan.

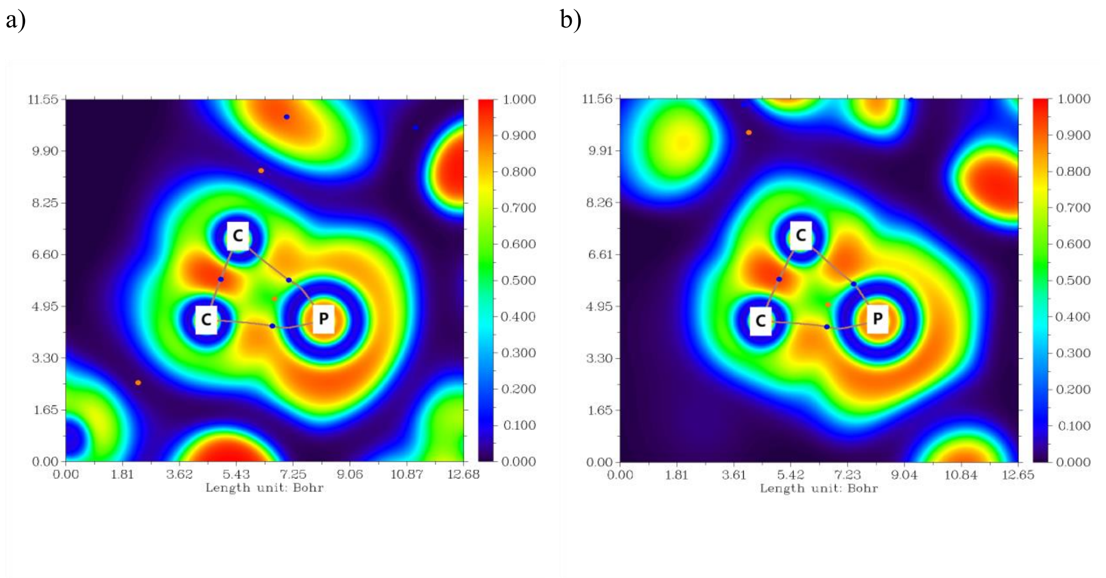

Supplementary Fig. 3 ELF plot. (a) the C_2P plane of **1**. (b) the C_2P plane of the phosphanorcaradiene reported by Stephan.

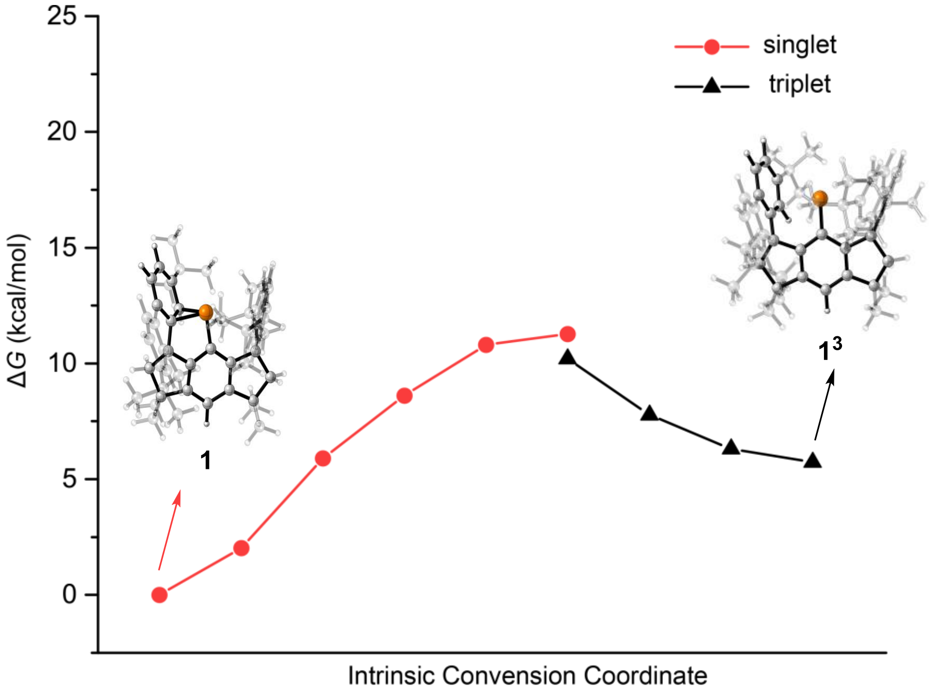

Supplementary Fig. 4 The interpolate analysis potential energy surface for the interconversion between **1** and triplet phosphinidene ($\mathbf{1}^3$ in the Fig.).

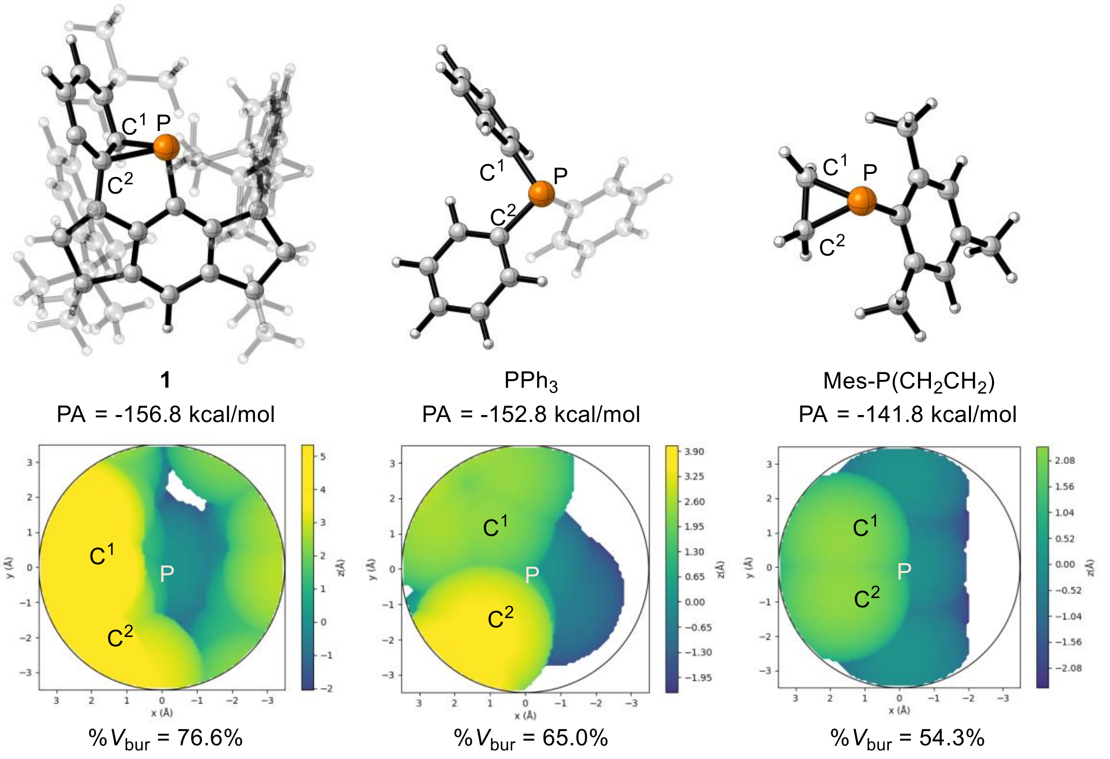

Supplementary Fig. 5 Analysis on proton affinity and steric effect of **1**, PPh_3 and Mes-P(CH_2CH_2).

Selected NMR spectra of 1-10

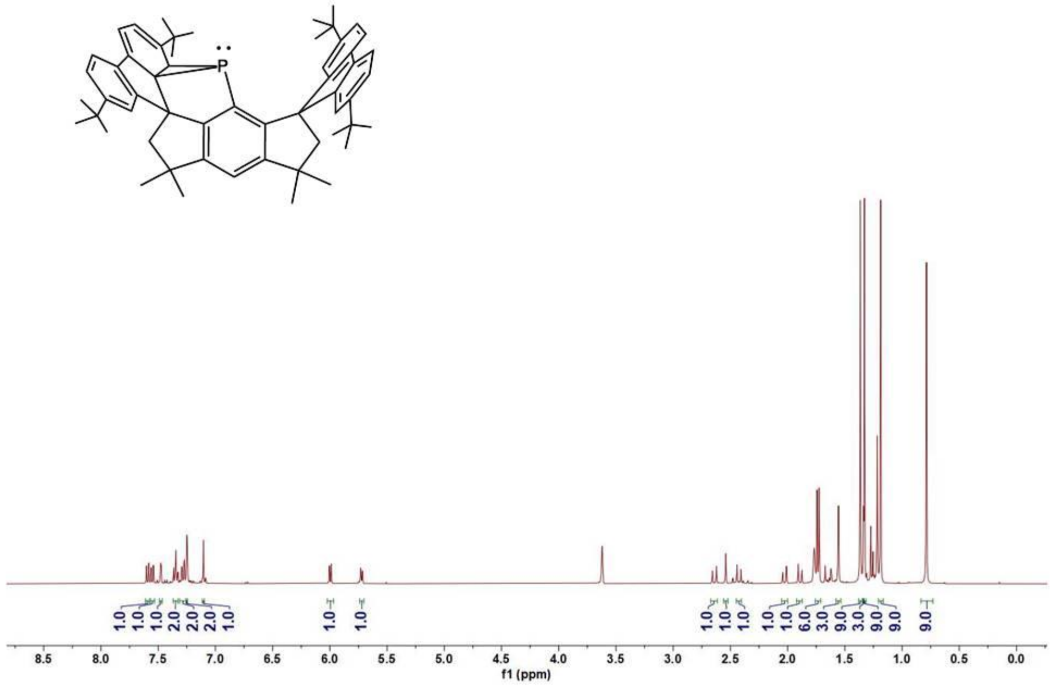

Supplementary Fig. 6 ^{1}H NMR spectrum of **1** in THF-d_8 at room temperature.

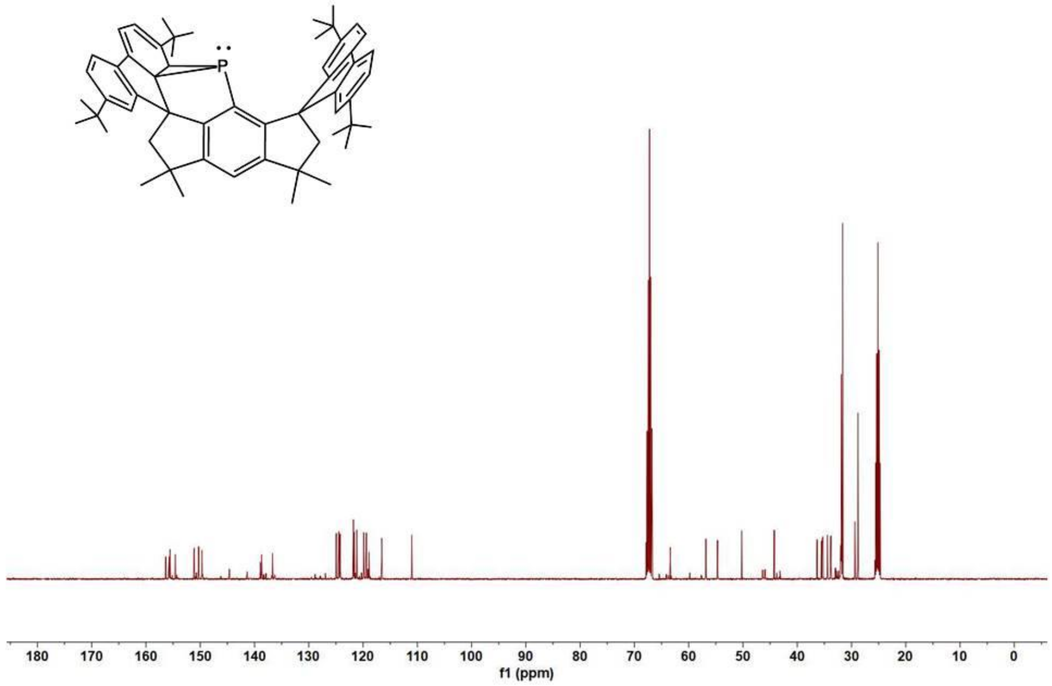

Supplementary Fig. 7 ^{13}C{^{1}H} NMR spectrum of **1** in THF-d_8 at room temperature.

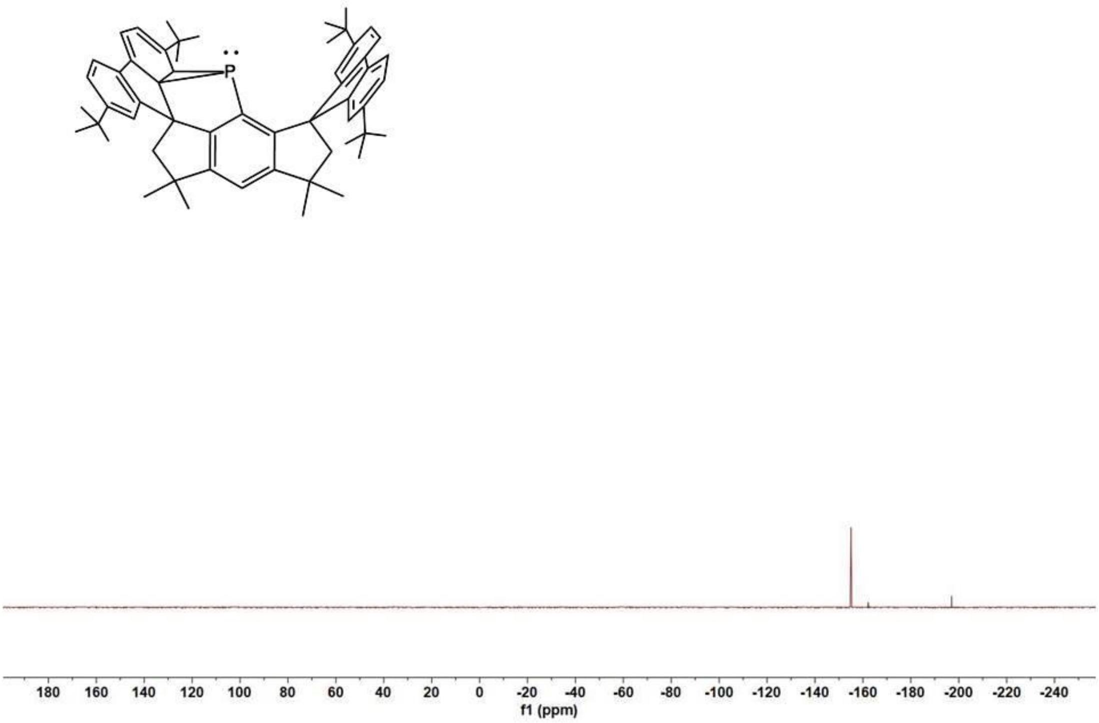

Supplementary Fig. 8 $^{31}P\{^{1}H\}$ NMR spectrum of **1** in THF-d_8 at room temperature.

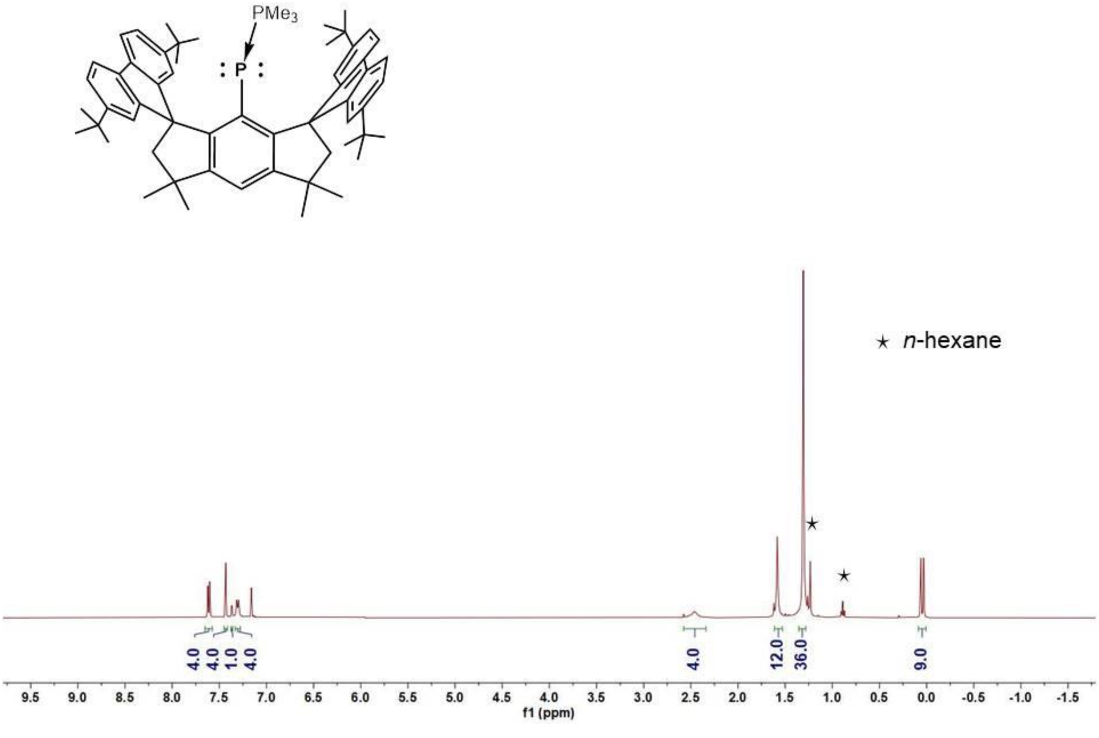

Supplementary Fig. 9 ^{1}H NMR spectrum of **2** in C_6D_6 at room temperature.

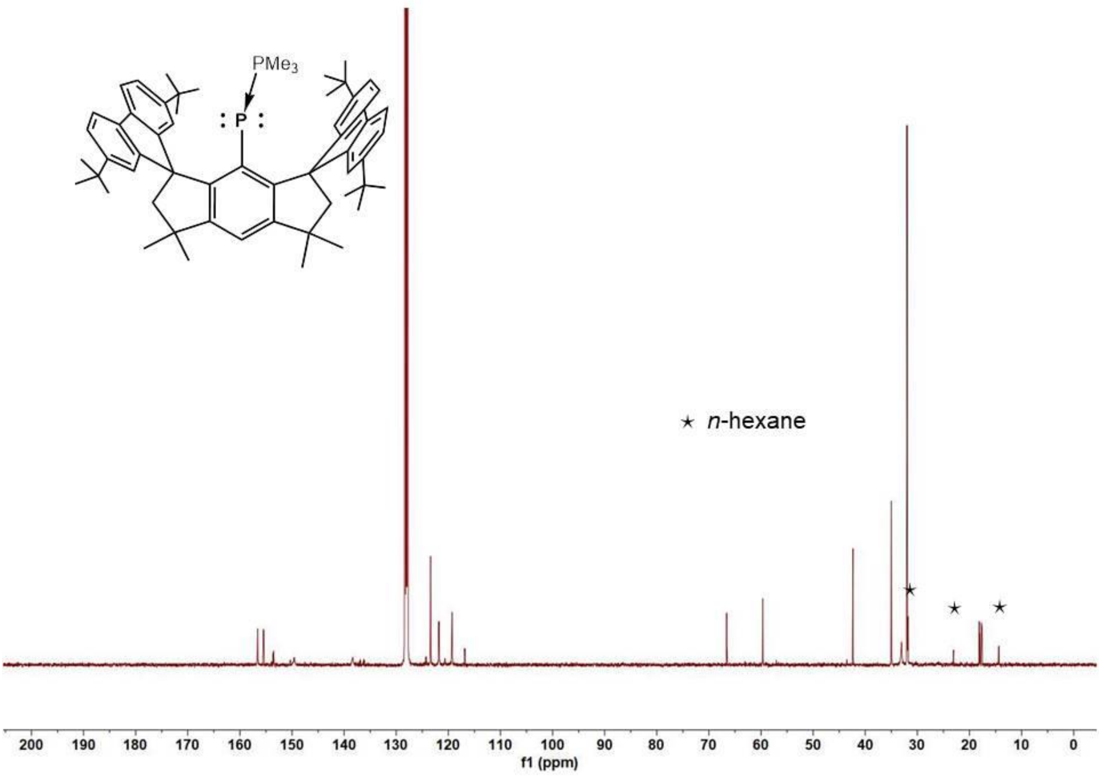

Supplementary Fig. 10 $^{13}C\{^{1}H\}$ NMR spectrum of **2** in C_6D_6 at room temperature.

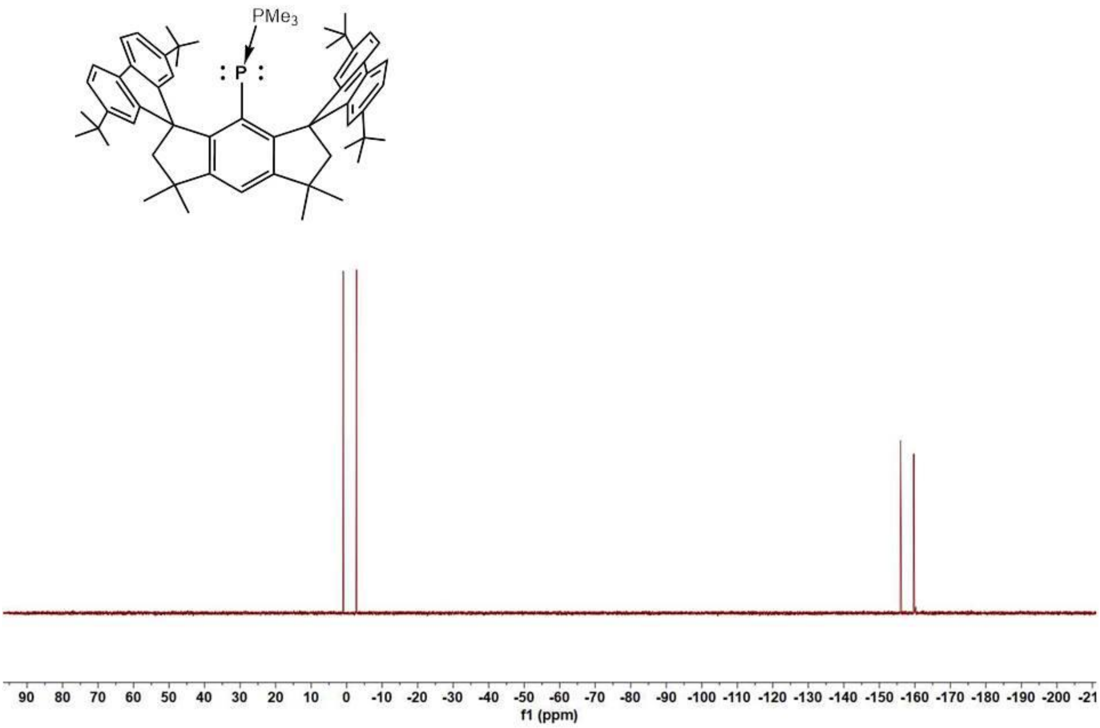

Supplementary Fig. 11 $^{31}P\{^{1}H\}$ NMR spectrum of **2** in C_6D_6 at room temperature.

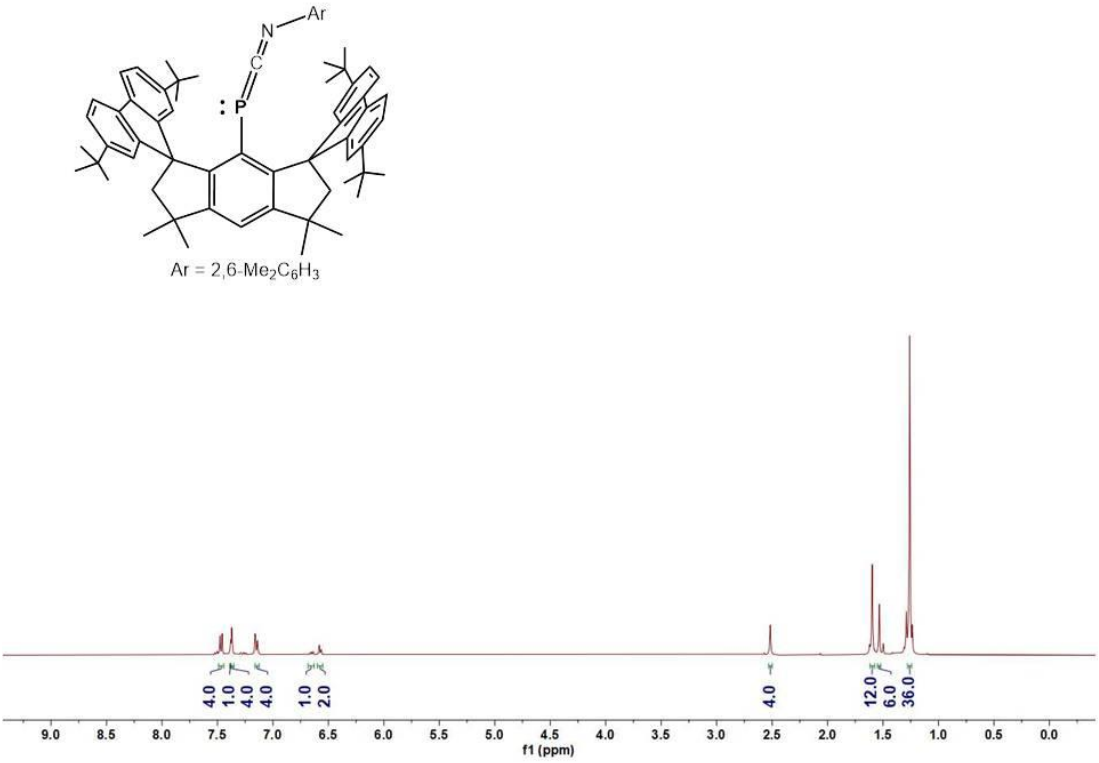

Supplementary Fig. 12 ^{1}H NMR spectrum of **3** in C_6D_6 at room temperature.

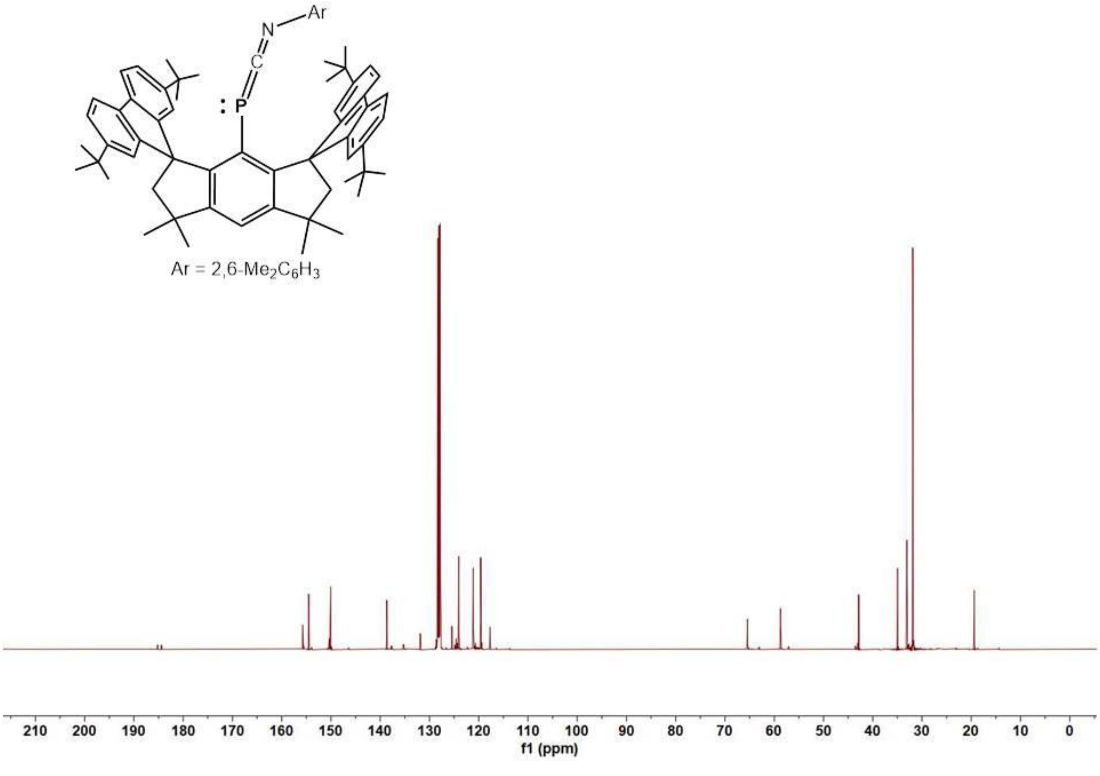

Supplementary Fig. 13 $^{13}C\{^{1}H\}$ NMR spectrum of **3** in C_6D_6 at room temperature.

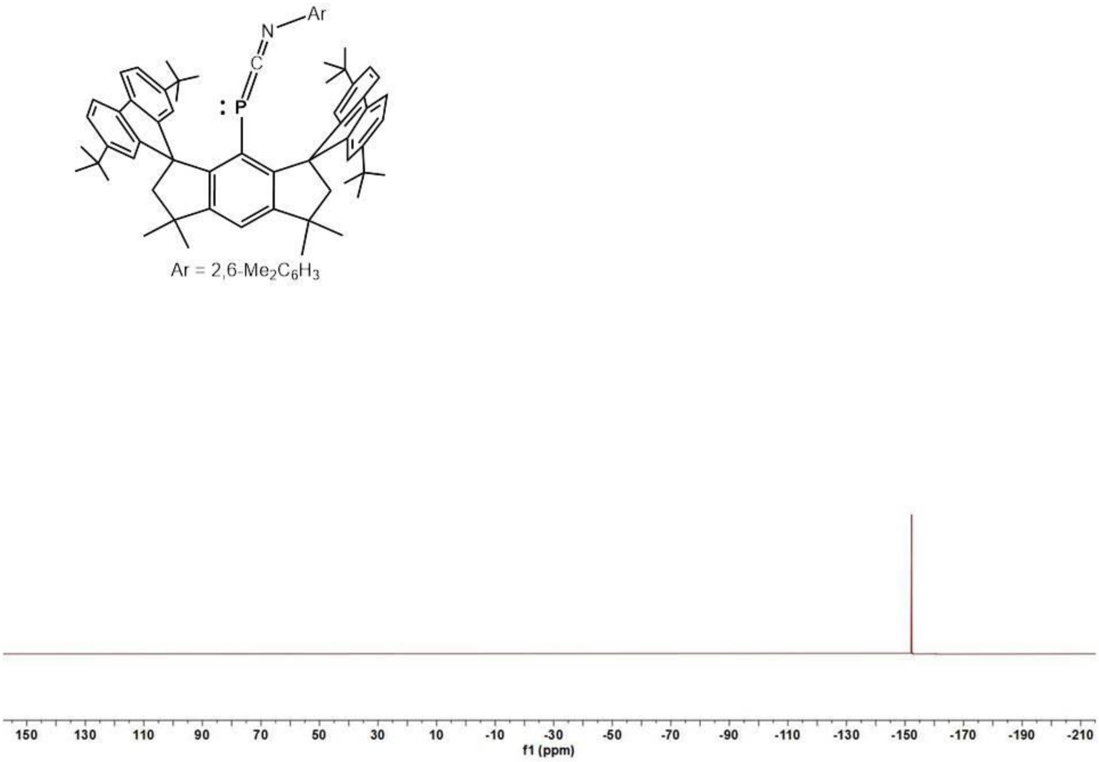

Supplementary Fig. 14 $^{31}P\{^{1}H\}$ NMR spectrum of **3** in C_6D_6 at room temperature.

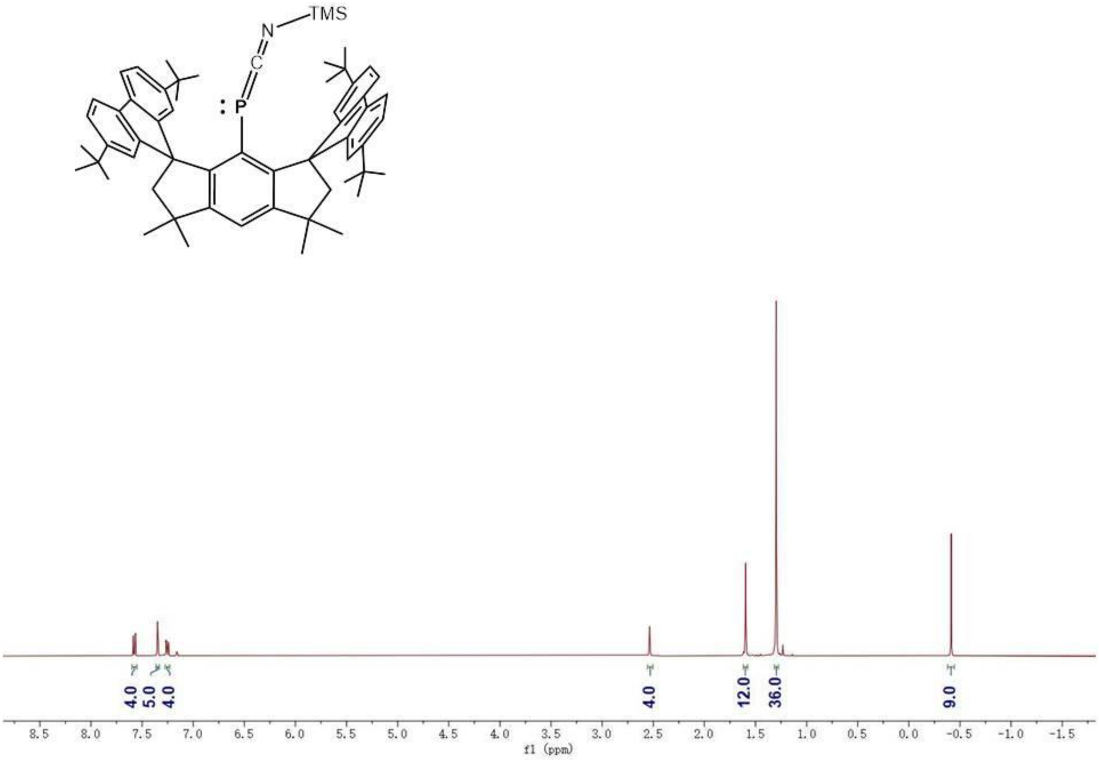

Supplementary Fig. 15 ^{1}H NMR spectrum of **4** in C_6D_6 at room temperature.

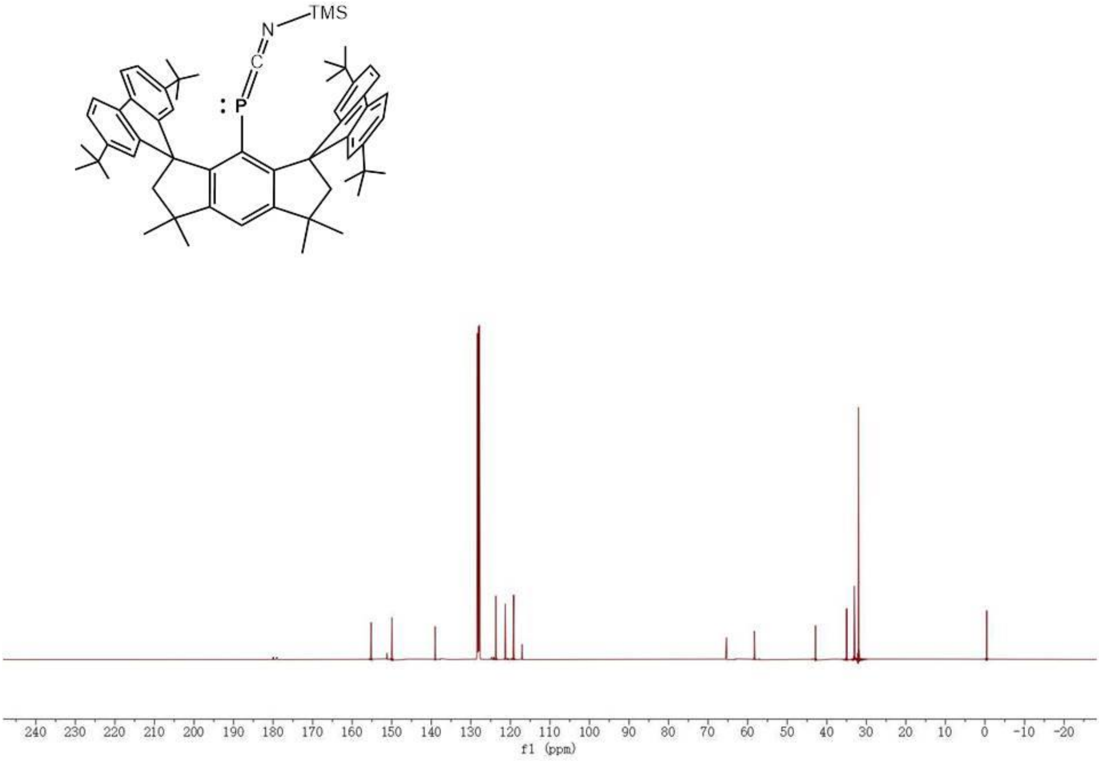

Supplementary Fig. 16 $^{13}C\{^{1}H\}$ NMR spectrum of **4** in C_6D_6 at room temperature.

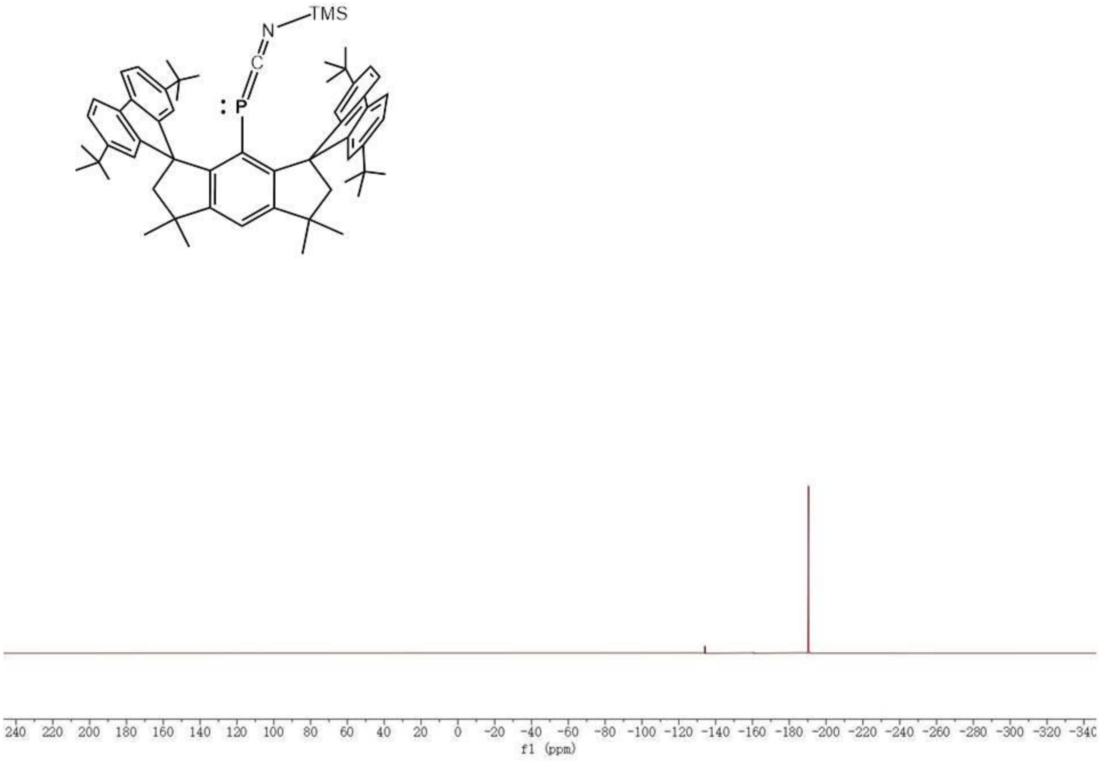

Supplementary Fig. 17 $^{31}P\{^{1}H\}$ NMR spectrum of **4** in C_6D_6 at room temperature.

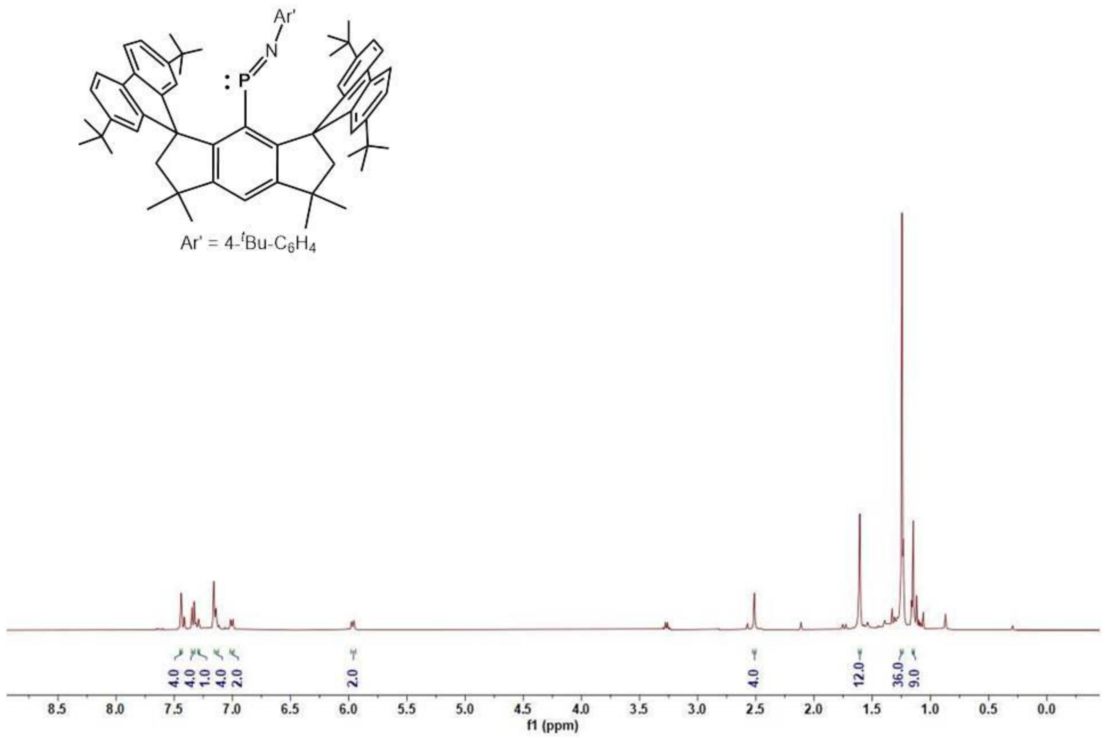

Supplementary Fig. 18 ^{1}H NMR spectrum of **5** in C_6D_6 at room temperature.

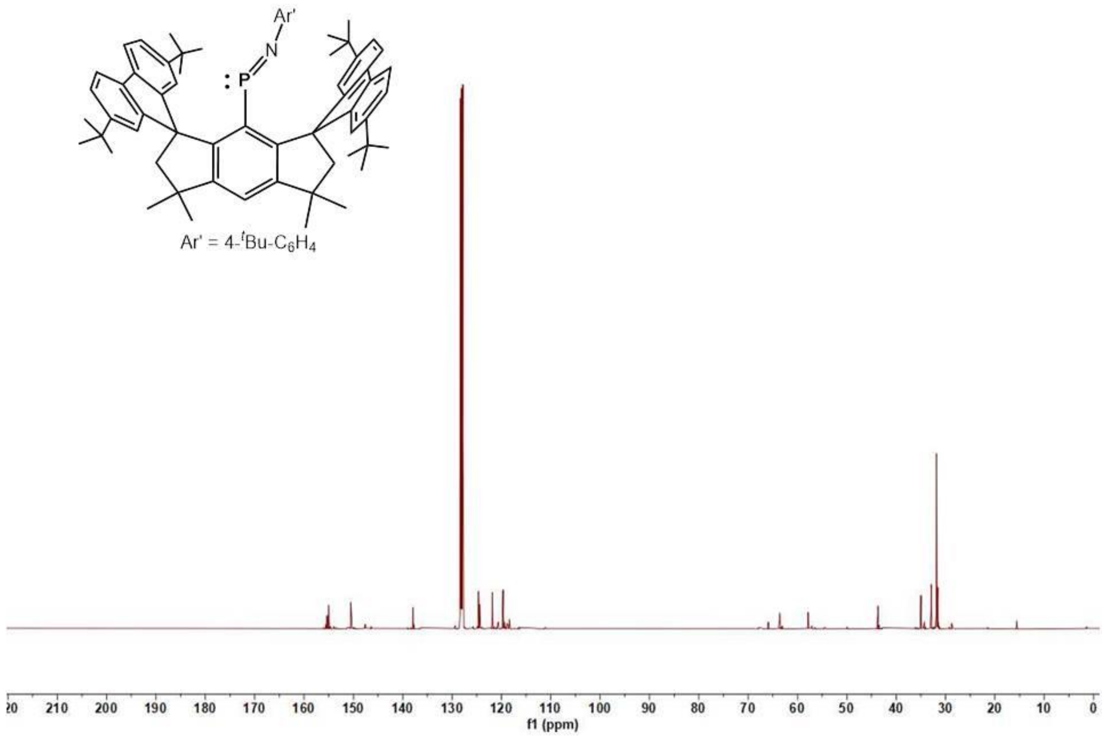

Supplementary Fig. 19 $^{13}C\{^{1}H\}$ NMR spectrum of **5** in C_6D_6 at room temperature.

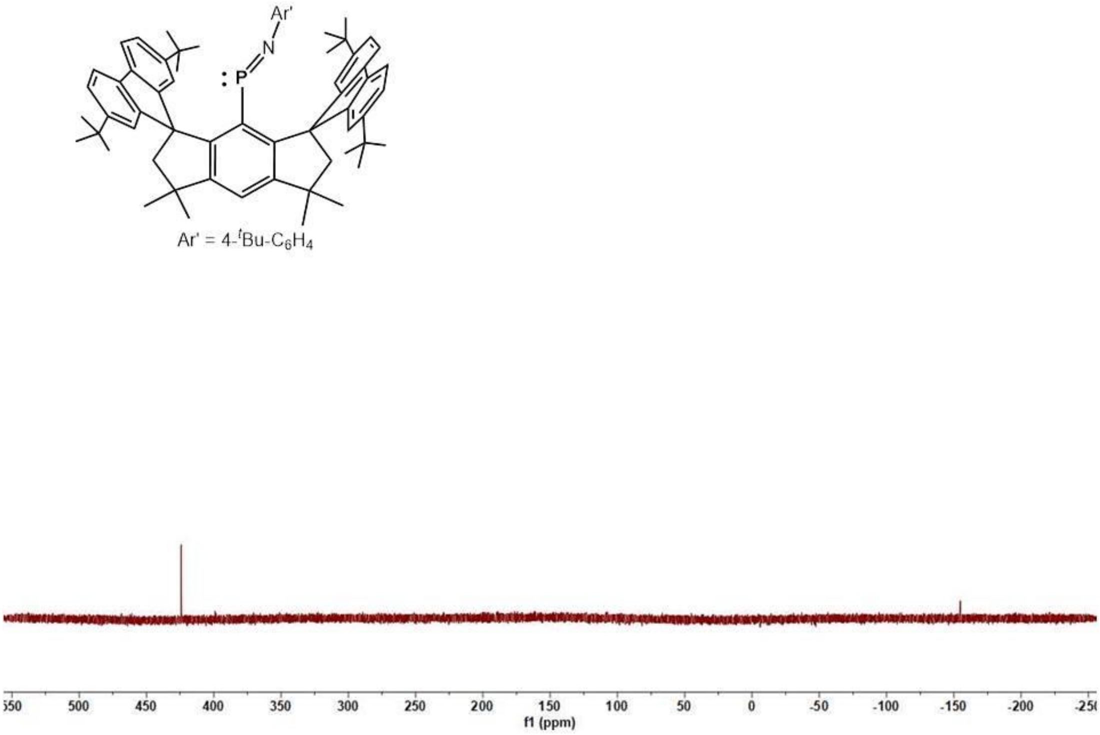

Supplementary Fig. 20 $^{31}P\{^1H\}$ NMR spectrum of **5** in C_6D_6 at room temperature.

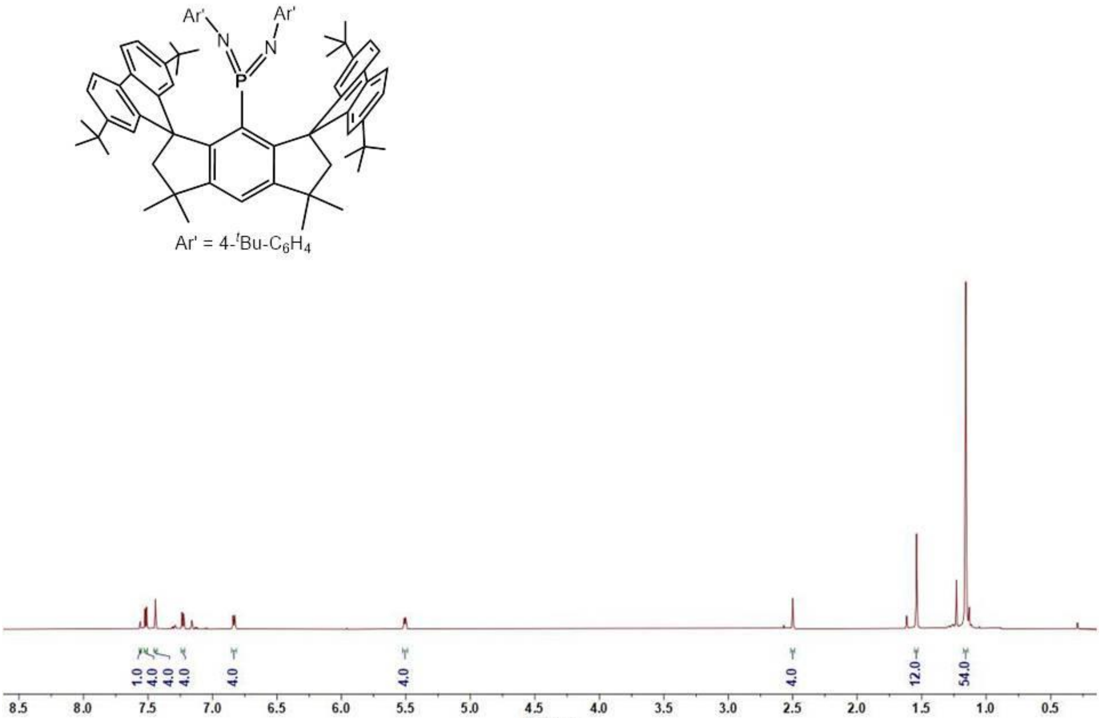

Supplementary Fig. 21 ^1H NMR spectrum of **6** in C_6D_6 at room temperature.

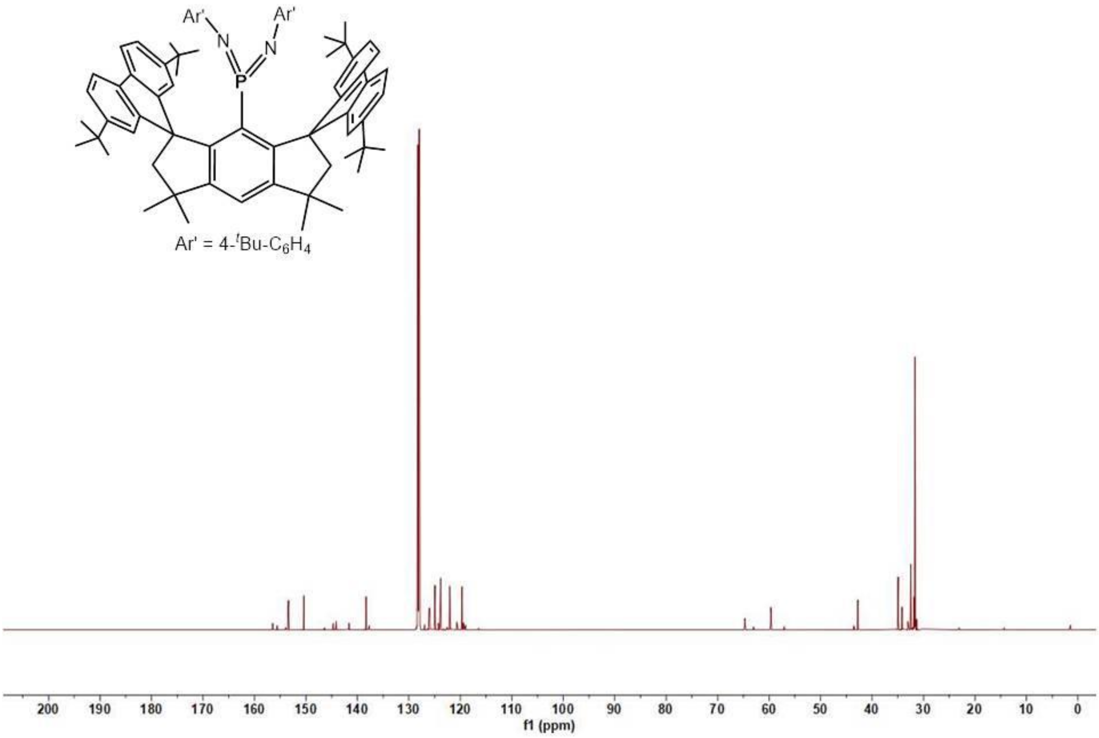

Supplementary Fig. 22 $^{13}C\{^1H\}$ NMR spectrum of **6** in C_6D_6 at room temperature.

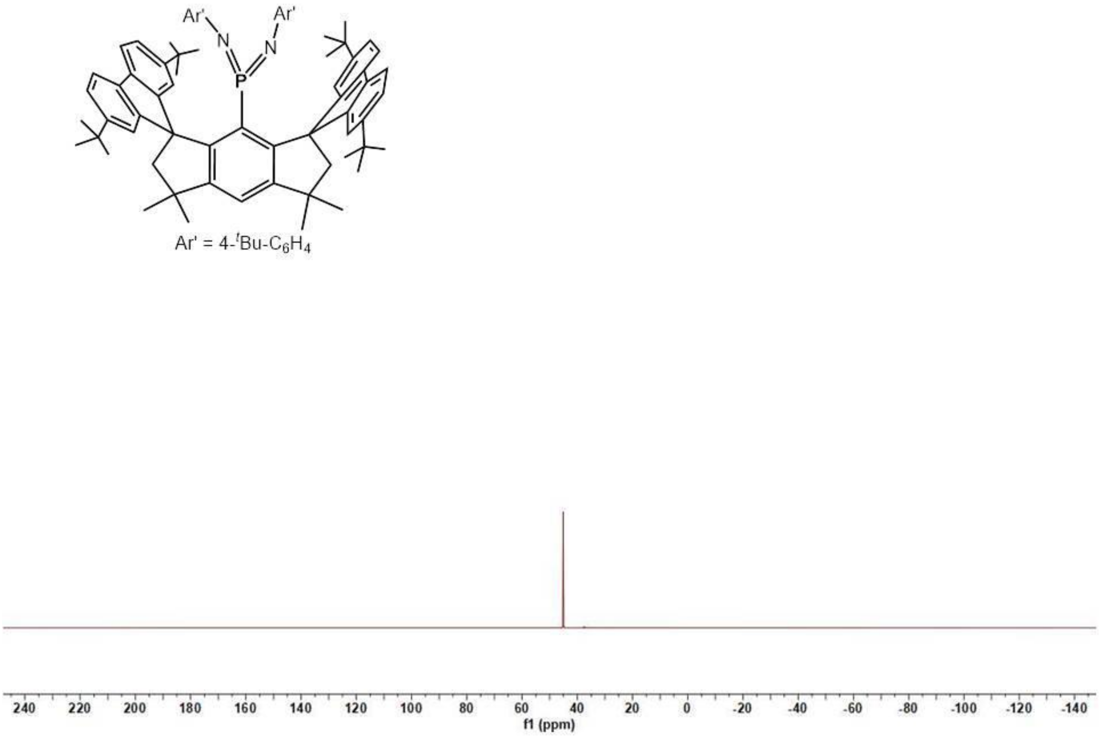

Supplementary Fig. 23 $^{31}P\{^1H\}$ NMR spectrum of **6** in C_6D_6 at room temperature.

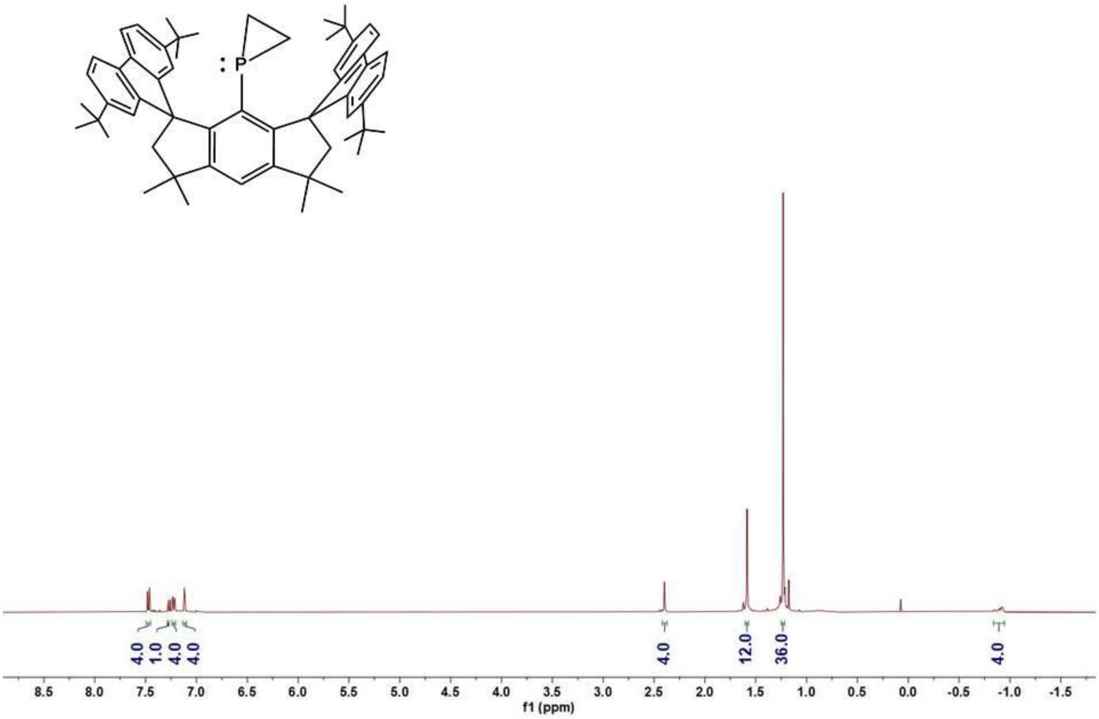

Supplementary Fig. 24 ^{1}H NMR spectrum of **7** in $CDCl_3$ at room temperature.

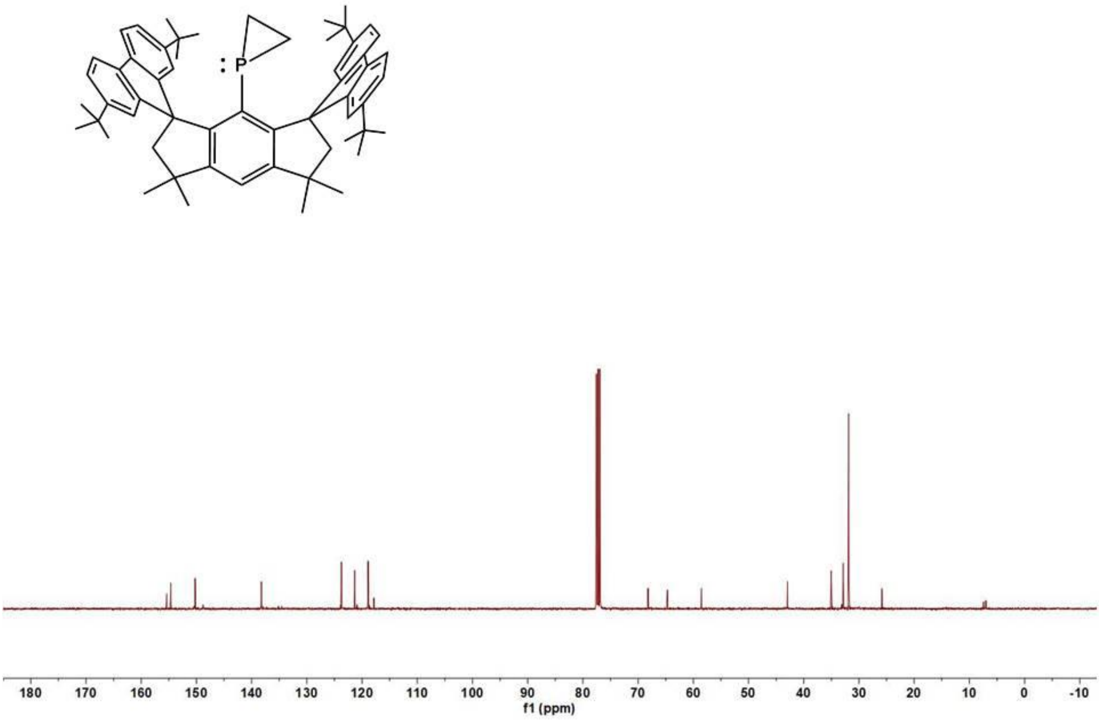

Supplementary Fig. 25 $^{13}C\{^{1}H\}$ NMR spectrum of **7** in $CDCl_3$ at room temperature.

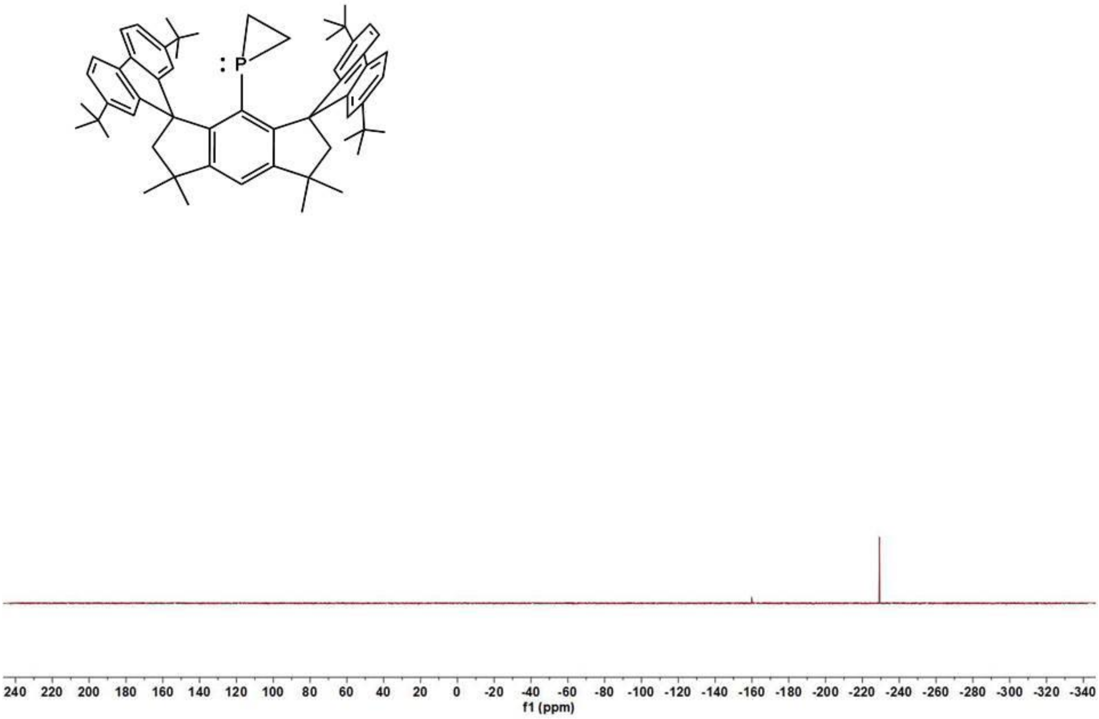

Supplementary Fig. 26 $^{31}P\{^{1}H\}$ NMR spectrum of **7** in $CDCl_3$ at room temperature.

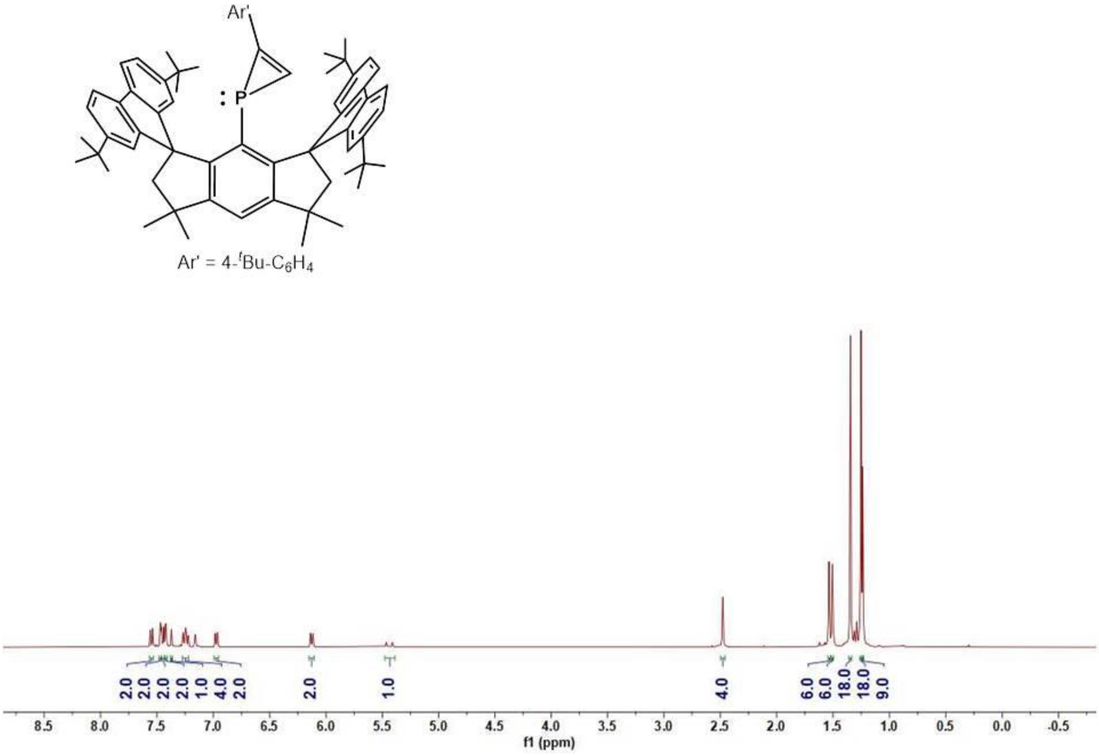

Supplementary Fig. 27 ^{1}H NMR spectrum of **8** in C_6D_6 at room temperature.

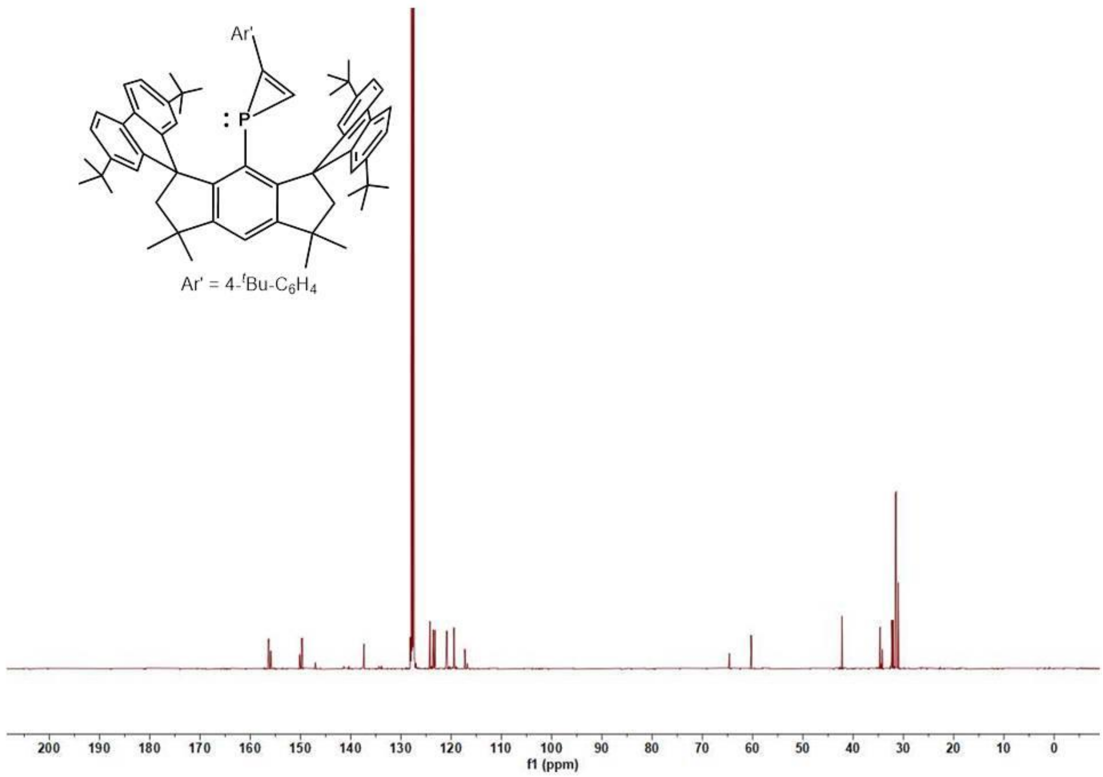

Supplementary Fig. 28 $^{13}C\{^{1}H\}$ NMR spectrum of **8** in C_6D_6 at room temperature.

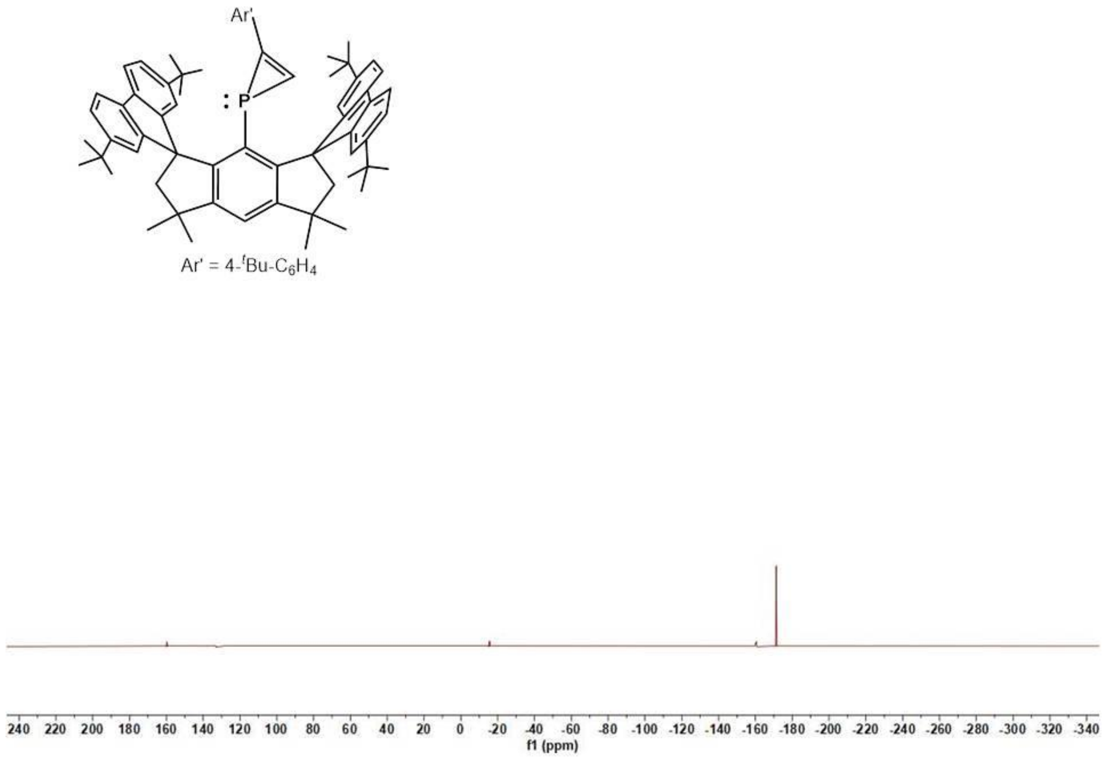

Supplementary Fig. 29 $^{31}P\{^{1}H\}$ NMR spectrum of **8** in C_6D_6 at room temperature.

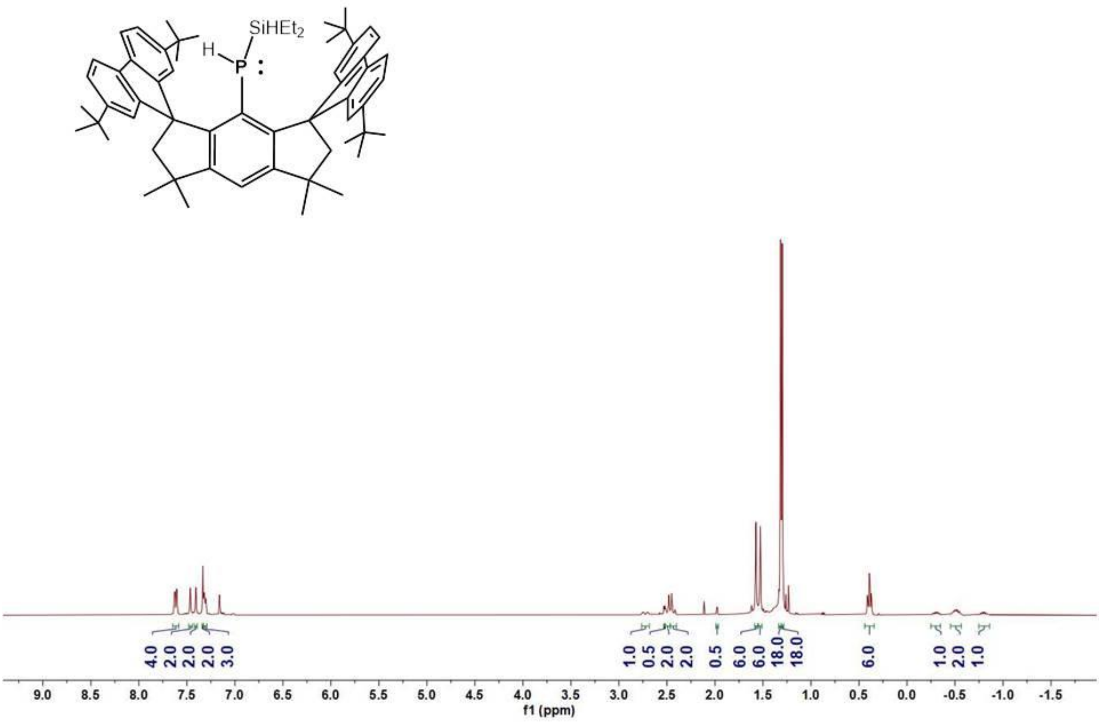

Supplementary Fig. 30 ^{1}H NMR spectrum of **9** in C_6D_6 at room temperature.

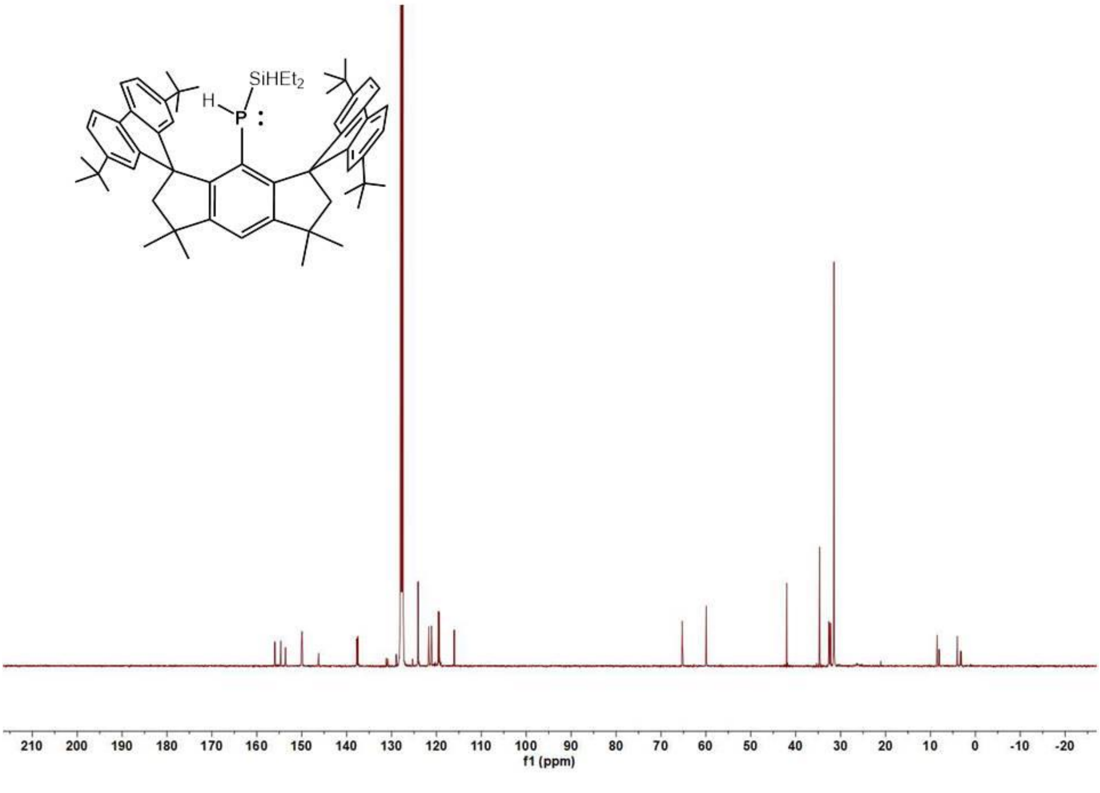

Supplementary Fig. 31 $^{13}C\{^{1}H\}$ NMR spectrum of **9** in C_6D_6 at room temperature.

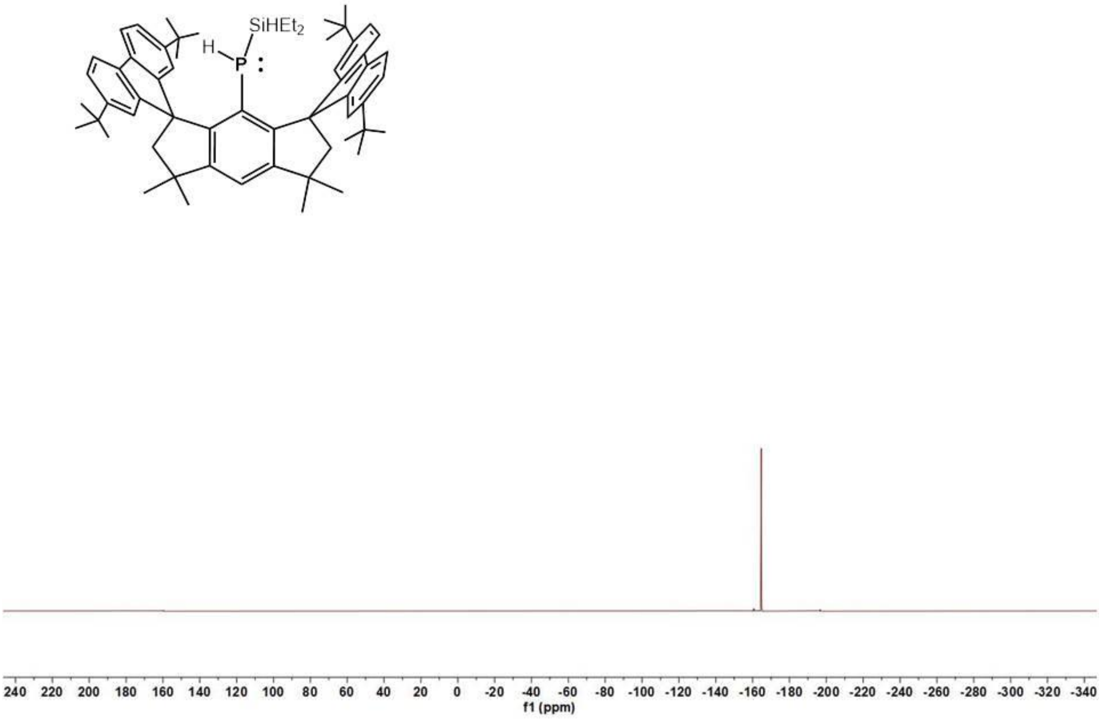

Supplementary Fig. 32 $^{31}P\{^{1}H\}$ NMR spectrum of **9** in C_6D_6 at room temperature.

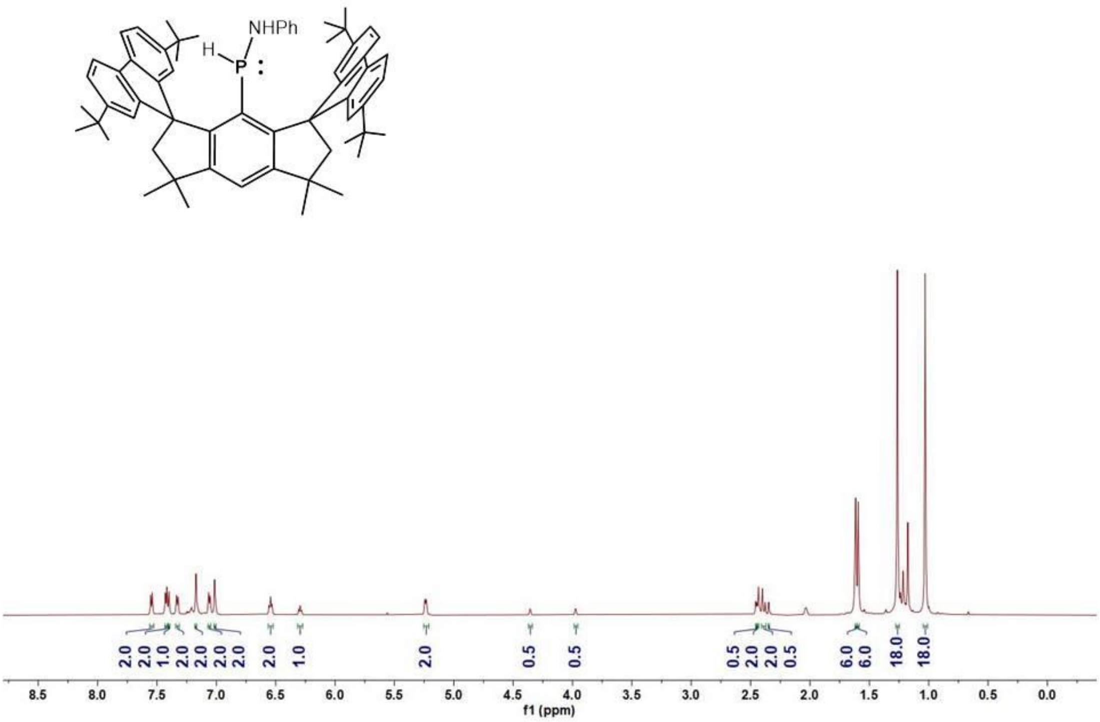

Supplementary Fig. 33 ^{1}H NMR spectrum of **10** in $CDCl_3$ at room temperature.

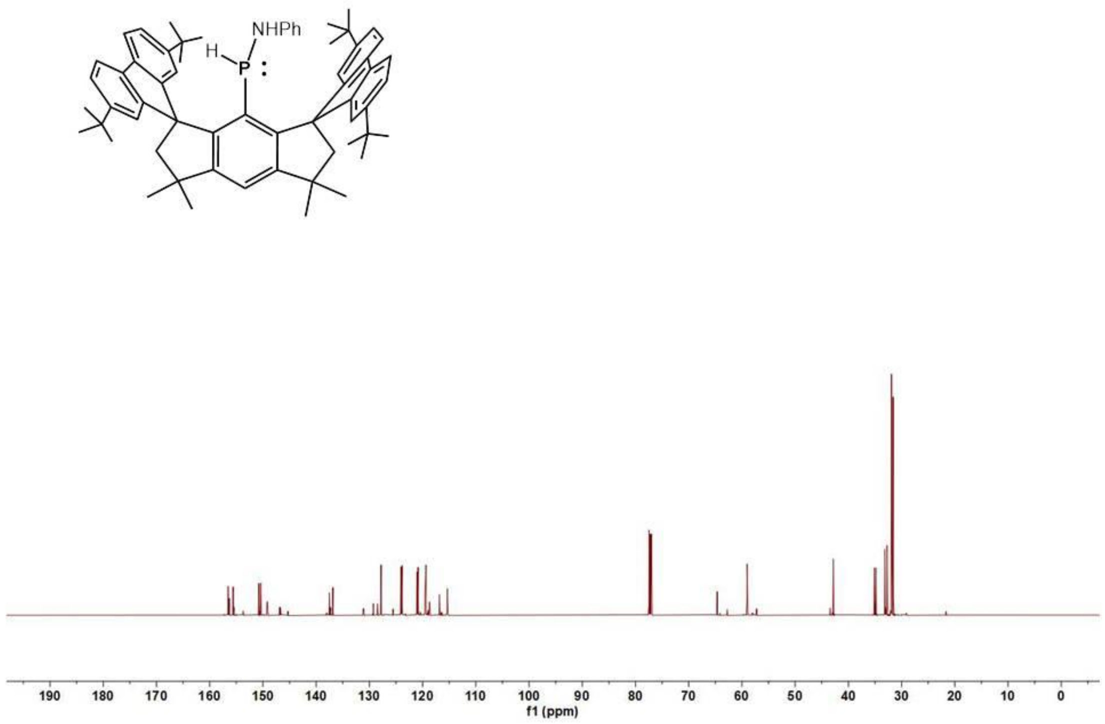

Supplementary Fig. 34 $^{13}C\{^{1}H\}$ NMR spectrum of **10** in $CDCl_3$ at room temperature.

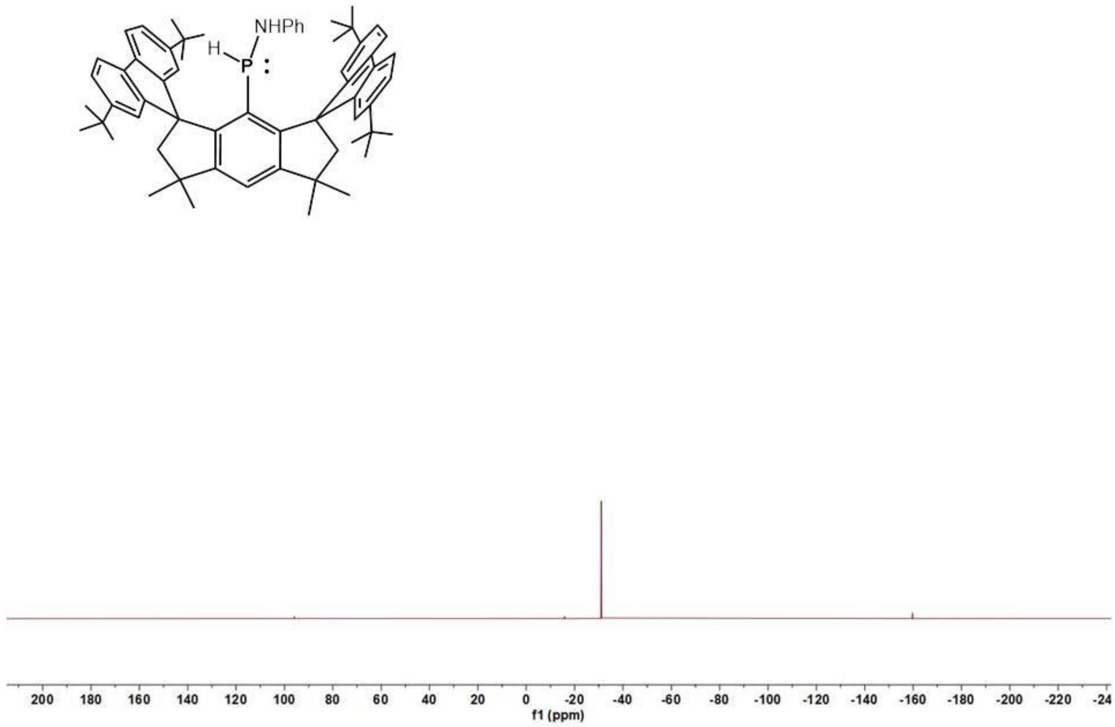

Supplementary Fig. 35 $^{31}P\{^{1}H\}$ NMR spectrum of **10** in $CDCl_3$ at room temperature.

References

[S1] Y. Chen, M. Wu, D. Wang, N. Yuan, L. Xu, G. Tan, *Organometallics* **2023**, *42*, 538-542.

[S2] D. M. Ottmers, H. F. Rase, *Carbon* **1966**, *4*, 125-127.

[S3] O. V. Dolomanov, L. J. Bourhis, R. J. Gildea, J. A. K. Howard, H. Puschmann, *J. App. Cryst.* **2009**, *42*, 339-341.

[S4] Bourhis, L.J., Dolomanov, O.V., Gildea, R.J., Howard, J.A.K., Puschmann, H. (2015). Acta Cryst. A71, 59-75.

[S5] G. Sheldrick, *Acta. Cryst. A* **2015**, *A71*, 3-8.

[S6] A. Spek, *Acta. Cryst.* **2015**, *C71*, 9-18.

[S7] A. D. Becke, *Phys. Rev. A* **1988**, *38*, 3098-3100.

[S8] J. P. Perdew, *Phys. Rev. B* **1986**, *33*, 8822-8824.

[S9] S. Grimme, S. Ehrlich, L. Goerigk, *J. Comp. Chem.* **2011**, *32*, 1456-1465.

[S10] F. Weigend, *Phys. Chem. Chem. Phys.* **2006**, *8*, 1057-1065.

[S11] F. Weigend, R. Ahlrichs, *Phys. Chem. Chem. Phys.* **2005**, *7*, 3297-3305.

[S12] A. V. Marenich, C. J. Cramer, D. G. Truhlar, *J. Phys. Chem. B* **2009**, *113*, 6378-6396.

[S13] M. J. Frisch, G. W. Trucks, H. B. Schlegel, G. E. Scuseria, M. A. Robb, J. R. Cheeseman, G. Scalmani, V. Barone, G. A. Petersson, H. Nakatsuji, X. Li, M. Caricato, A. V. Marenich, J. Bloino, B. G. Janesko, R. Gomperts, B. Mennucci, H. P. Hratchian, J. V. Ortiz, A. F. Izmaylov, J. L. Sonnenberg, D. Williams-Young, F. Ding, F. Lipparini, F. Egidi, J. Goings, B. Peng, A. Petrone, T. Henderson, D. Ranasinghe, V. G. Zakrzewski, J. Gao, N. Rega, G. Zheng, W. Liang, M. Hada, M. Ehara, K. Toyota, R. Fukuda, J. Hasegawa, M. Ishida, T. Nakajima, Y. Honda, O. Kitao, H. Nakai, T. Vreven, K. Throssell, J. A. Montgomery, Jr., J. E. Peralta, F. Ogliaro, M. J. Bearpark, J. J. Heyd, E. N. Brothers, K. N. Kudin, V. N. Staroverov, T. A. Keith, R. Kobayashi, J. Normand, K. Raghavachari, A. P. Rendell, J. C. Burant, S. S. Iyengar, J. Tomasi, M. Cossi, J. M. Millam, M. Klene, C. Adamo, R. Cammi, J. W. Ochterski, R. L. Martin, K. Morokuma, O. Farkas, J. B. Foresman, D. J. Fox, *Gaussian 16*, Revision C.01, Gaussian, Inc., Wallingford CT, **2019**.

[S14] Legault, C. Y. CYLview, Université de Sherbrooke, Québec, **2009**, http://www.cylview.org/.

[S15] a) A. D. Becke, K. E. Edgecombe, *J. Chem. Phys.* **1990**, *92*, 5397-5403; b) A. Savin, R. Nesper, S. Wengert, T. F. Fässler, *Angew. Chem. Int. Ed.* **1997**, *36*, 1808-1832. c) C. F. Matta, R. J. Boyd, *The Quantum Theory of Atoms in Molecules*, Wiley-VCH, Weinheim, **2007**.

[S16] T. Lu, F. Chen, *Acta Phys. -Chim. Sin.* **2011**, *27*, 2786-2792.

[S17] T. Lu, F. Chen, *J. Comput. Chem.* **2012**, *33*, 580-592.

[S18] Y. Zhao, D. G. Truhlar, *Theor. Chem. Acc.* **2008**, *120*, 215-241. A. E. Reed, L. A. Curtiss, F. Weinhold, *Chem. Rev.* **1988**, *88*, 899-926.

[S19] a) G. Knizia, *J. Chem. Theory Comput.* **2013**, *9*, 4834-4843; b) G. Knizia, J. E. M. N. Klein, *Angew. Chem. Int. Ed.* **2015**, *54*, 5518-5522.

[S20] F. Neese, *WIREs Comput. Mol. Sci.* **2012**, *2*, 73-78.

[S21] K. Jorner, *MORFEUS*, **2021**, https://digital-chemistry-laboratory.github.io/morfeus/index.html#.

[S22] L. Falivene, Z. Cao, A. Petta, L. Serra, A. Poater, R. Oliva, V. Scarano, L. Cavallo, *Nat. Chem.* **2019**, *11*, 872-879.
